# A multi-marker integrative analysis reveals benefits and risks of bariatric surgery

**DOI:** 10.1038/s41598-022-23241-6

**Published:** 2022-11-07

**Authors:** Simonetta Palleschi, Valeria Guglielmi, Lorenza Nisticò, Carla Ferreri, Claudio Tabolacci, Francesco Facchiano, Egidio Iorio, Alessandro Giuliani, Sonia Brescianini, Emanuela Medda, Corrado Fagnani, Barbara Rossi, Anna Minoprio, Mattea Chirico, Maria Elena Pisanu, Federica Di Nolfo, Paola Fortini, Valeria Simonelli, Sara Baccarini, Serena Laterza, Tiziana Morretti, Ambra Dell’Orso, Federico Manganello, Paolo Gentileschi, Paolo Sbraccia, Eugenia Dogliotti

**Affiliations:** 1 Department of Environment and Health, Istituto Superiore di Sanità, Viale Regina Elena 299, 00161 Rome, Italy; 2 Internal Medicine Unit and Obesity Center, University Hospital Policlinico Tor Vergata, 00133 Rome, Italy; 3 Department of Systems Medicine, University of Rome Tor Vergata, 00133 Rome, Italy; 4 Center for Behavioral Sciences and Mental Health, Istituto Superiore di Sanità, 00161 Rome, Italy; 5 Consiglio Nazionale delle Ricerche, ISOF, 40129 Bologna, Italy; 6 Department of Oncology and Molecular Medicine, Istituto Superiore di Sanità, 00161 Rome, Italy; 7 Core Facilities, Istituto Superiore di Sanità, 00161 Rome, Italy; 8 Lipidomic Laboratory, Lipinutragen Srl, 40128 Bologna, Italy; 9 Bariatric Surgery Unit, Department of Surgery, University of Rome Tor Vergata, 00133 Rome, Italy

**Keywords:** Computational biology and bioinformatics, Physiology, Biomarkers, Endocrinology

## Abstract

Bariatric surgery (BS) is an effective intervention for severe obesity and associated comorbidities. Although several studies have addressed the clinical and metabolic effects of BS, an integrative analysis of the complex body response to surgery is still lacking. We conducted a longitudinal data study with 36 patients with severe obesity who were tested before, 6 and 12 months after restrictive BS for more than one hundred blood biomarkers, including clinical, oxidative stress and metabolic markers, peptide mediators and red blood cell membrane lipids. By using a synthetic data-driven modeling based on principal component and correlation analyses, we provided evidence that, besides the early, well-known glucose metabolism- and weight loss-associated beneficial effects of BS, a tardive, weight-independent increase of the hepatic cholesterol metabolism occurs that is associated with potentially detrimental inflammatory and metabolic effects. Canonical correlation analysis indicated that oxidative stress is the most predictive feature of the BS-induced changes of both glucose and lipids metabolism. Our results show the power of multi-level correlation analysis to uncover the network of biological pathways affected by BS. This approach highlighted potential health risks of restrictive BS that are disregarded with the current practice to use weight loss as surrogate of BS success.

## Introduction

Obesity is a growing problem around the world and Europe is not an exception. Italy is among the European countries with the lowest prevalence among adults (10%) but with two out of five children overweight or with obesity^1,2^. Obesity is overtaking smoking as the leading cause of premature death and is associated with several debilitating and deadly diseases, the risk of which progressively increases with increasing Body Mass Index (BMI)^3^. A key risk factor for the development of serious medical illnesses in people with obesity is the abnormal accumulation of visceral fat, that is an endocrine organ of energy storage and producer of inflammatory adipokines, thus causing low-grade chronic inflammation. Concomitantly, fatty acids concentration increases in plasma due to the expansion of fat mass thus promoting the formation of highly reactive oxygen species and inducing oxidative stress^4,5^. Increased levels of pro-inflammatory cytokines and free radicals lead to insulin resistance that is a key etiological factor in the development of chronic non-communicable diseases such as type 2 diabetes and cardiovascular diseases^6^. Inflammation and alteration of hormones metabolism might also explain the association of obesity with risk of cancer at several organ sites, with severe obesity (BMI ≥ 40 kg/m^2^) and esophagus and *corpus uteri* showing the highest relative risk^7^.

While lifestyle modifications and pharmacological therapies are the cornerstones of weight management and prevention of cardiovascular risk factors, these are usually not enough to treat severe obesity. In this case bariatric surgery (BS) has become increasingly frequent by efficiently reducing weight excess and associated comorbidities (e.g. type 2 diabetes, obstructive sleep apnea, hypertension, dyslipidemia). However, the benefits of weight loss following bariatric procedures are still debated regarding the pro-inflammatory and metabolic profile of obesity^8^.

It is known that obesity affects several regulation pathways relative to different organization layers such as metabolic, endocrine, inflammatory, neural and cell-intrinsic pathways but an integrative analysis of the complex organism response to fat accumulation and its perturbation upon BS is still missing. The analysis of a large number of biomarkers in body fluids as well as in circulating cells by high-throughput technologies offers the opportunity to identify the obesity-associated pathways that may be regulated by BS and unravels the connections among them. Moreover, studying the dynamics of these pathways under conditions of weight loss offers new tools to address the need for diagnostic and even more prognostic markers to reduce the risk of obesity-associated comorbidities^9^.

In the present study, we exploit a data-driven, integrative analysis to investigate the dynamics of multiple blood biomarkers in a cohort of subjects with severe obesity undergoing BS to gain insights into the network of physiological changes induced by BS and the underlying mechanisms.

## Materials and methods

### Subjects and samples

Between 2017 and 2019 we enrolled 36 subjects with severe obesity (33 females; mean age 47.1 ± 10.8 years; mean BMI 44.3 ± 6.9 kg/m^2^) attending the outpatient service of the Obesity Center of the University Hospital ‘Policlinico Tor Vergata’ (Rome, Italy) for clinical evaluation before BS. Eligible patients met the criteria for BS and had a stable body weight in the last 3 months preceding the study. Exclusion criteria were as follows: known medical history of diabetes or self-reported use of hypoglycemic agents; chronic liver or kidney disease; infections; malignancy; other acute or chronic systemic diseases; use of glucocorticoids, non-steroidal anti-inflammatory medications or antibiotics in the last month prior to BS. All enrolled patients underwent laparoscopic BS, mostly restrictive procedures (sleeve gastrectomy 17 [47.2%]; banded sleeve gastrectomy 13 [36.1%]; adjustable gastric banding 1 [2.8%]; banded Roux-en Y gastric bypass 3 [8.3%]; mini-gastric bypass 2 [5.6%]). At enrollment and 6 and 12 months after surgery, all the participants underwent a comprehensive medical evaluation, including the physical examination and the collection of blood samples for biomarkers measurements. After surgery, patients received periodic counseling about dietary and lifestyle modifications as recommended^10^. Exams were carried out in the morning on fasted subjects. Blood samples were analyzed immediately (hematology and clinical biochemistry assays) or processed and stored in aliquots at either + 4 °C (whole blood) or − 80 °C (serum and plasma) until use. To obtain reference values for our biomarkers, we also enrolled 16 normal weight (NW) subjects (69% female; age 36.6 ± 11 years; BMI 23.5 ± 2, mean ± SD) who underwent—only once—the same clinical and biochemical assessment as bariatric patients. All NW subjects met the same exclusion criteria adopted for bariatric patients.

### Anthropometric, clinical and clinical biochemistry measurements

Anthropometric (weight, height, BMI, waist and hip circumference), clinical (blood pressure) and hematological and biochemical parameters were assessed as previously described^11,12^. Tumor necrosis factor-α (TNF-α) and Interleukin-6 serum levels were evaluated by ELISA using commercially available kits. Homeostatic model assessment for insulin resistance (HOMA-IR) was calculated according to the formula: fasting insulin (μU/L) × fasting glucose (mg/dl)/405. Metabolic syndrome was diagnosed according to International Diabetes Federation criteria^13^.

### Plasma oxidative stress markers

Plasma samples were analyzed for oxidative damage biomarkers (Advanced Oxidation Protein Products, total free thiols, total malondialdehyde), antioxidant and nutritional biomarkers (vitamin A and E, total proteins) and low-MW thiols related to glutathione metabolism and cellular response to oxidative stress (glutathione, cysteine, cysteinylglycine and homocysteine). Advanced Oxidation Protein Products, total free thiols and total proteins were determined by colorimetric methods^14,15^ adapted to an automatic biochemistry analyzer (ILAB300plus, Instrumentation Laboratory SpA, Milan, Italy). Total malondialdehyde, vitamins and low-MW thiols were measured by HPLC with UV or FL detection based on previously described methods^16–18^ with slight modifications^19–21^.

### ^1^H NMR-based serum metabolic markers

Intact serum samples, properly diluted with 0.9% NaCl in D_2_O, were analyzed at 298 K in 9.4 T Bruker Avance spectrometer at 400 MHz (Bruker, Karlsruhe, Germany)^22^. ^1^H NMR spectral analyses and signals quantification were performed according to established protocols set up in our laboratory^23^. Standard presaturation pulse sequence and spin echo Carr–Purcell–Meiboom–Gill 1D sequence^22^ were applied on each intact serum sample. Relative metabolite quantification was expressed as metabolite percentage relative to total metabolites investigated.

### Plasma peptide mediators

Plasma cytokines/chemokines and other circulating proteins levels were measured by xMAP technology on a X200 Luminex platform (Bio-Plex 200 System, Bio-Rad Laboratories, Inc., Hercules, CA, USA) equipped with a magnetic workstation (see Supplementary Table S1 for the full list). All peptides, with the exception of TNF-α (TNF-αLum), were determined using Human Magnetic Luminex Performance Assay kits (R&D Systems, Minneapolis, MN, USA). TNF-αLum was measured using a Human High Sensitivity Magnetic Luminex Performance Assay kit (R&D Systems). Data were analyzed by Bio-Plex Manager software (version 6.1, Bio-Rad Laboratories, Inc.) and results were expressed as pg/ml or ng/ml. Adiponectin and vascular endothelial growth factor-C (VEGF-C) plasma levels were evaluated by ELISA (R&D Systems kit) according to manufacturer’s instructions.

### Red blood cell membrane fatty acids

RBC membrane FA analysis was performed by gas chromatography/flame ionization detector as previously described^24^. Briefly, the blood samples (550 µL) in EDTA as anticoagulant were checked for the absence of hemolysis and treated by an automated procedure to isolate the mature RBC fraction and the membrane lipid pellet, to extract lipids and perform trans-esterification to fatty acid methyl ester. A pool of 16 FAs was selected as a representative profile of the dominant glycerophospholipids present in the RBC membrane (see Supplementary Table S1 for the full list). All of them were recognized by standard references as previously described^25,26^.

### Statistical analyses

Collectively, 105 variables were measured at each of the three time points, and used for bioinformatics analysis. All continuous variables were summarised using medians with interquartile ranges or means with standard deviations. Mann–Whitney test was applied for comparisons of biomarkers levels between patients and normal-weight subjects at baseline, while Wilcoxon matched-pairs signed-rank test was applied to investigate changes during the follow-up. The significance level was set at p < 0.05 for all comparisons; in this respect, due to the exploratory nature of this study, and the small sample size, neither Bonferroni correction nor False Discovery Rate (FDR) were applied. The biomarkers data of all subjects at the three time points of the study were subjected to independent principal component analyses (PCAs) in order to extract the main correlation fluxes shaping each data set^27^ (see Supplementary material for details). The extracted components were then assigned a biological meaning by the inspection of their loading (correlation coefficient between original variables and components) pattern^27^. The component scores were in turn analyzed by repeated-measures Analysis of Variance (ANOVA) to check for the presence of a statistically significant relation between components and BS follow-up. Spearman correlation analysis was applied to assess the correlation degree between PCs from different local datasets.

In a multi-level perspective, the local analyses were each other correlated, by means of a canonical correlation strategy^28,29^. The statistically significant canonical correlations allowed generation of a network having as nodes the different local data sets and as edges the presence of a significant canonical correlation, so providing an integrative multi-level perspective on the different features of BS^30,31^. All data were analyzed using Stata statistical software (StataCorp. 2019. *Stata Statistical Software*: Release 16. College Station, TX: StataCorp LLC; https://www.stata.com).

### Ethical approval

The study was conducted according to the guidelines of the Declaration of Helsinki, and approved by the Ethical Committees of the Fondazione Policlinico Tor Vergata, Rome (n. 169/15, 18 December 2015) and of the Istituto Superiore di Sanità (n. 173/16, 15 March 2016). Written informed consent was obtained from each participant before inclusion in the study.

## Results

### Dynamics of clinical and metabolic changes across time after bariatric surgery

**Figure 1:**
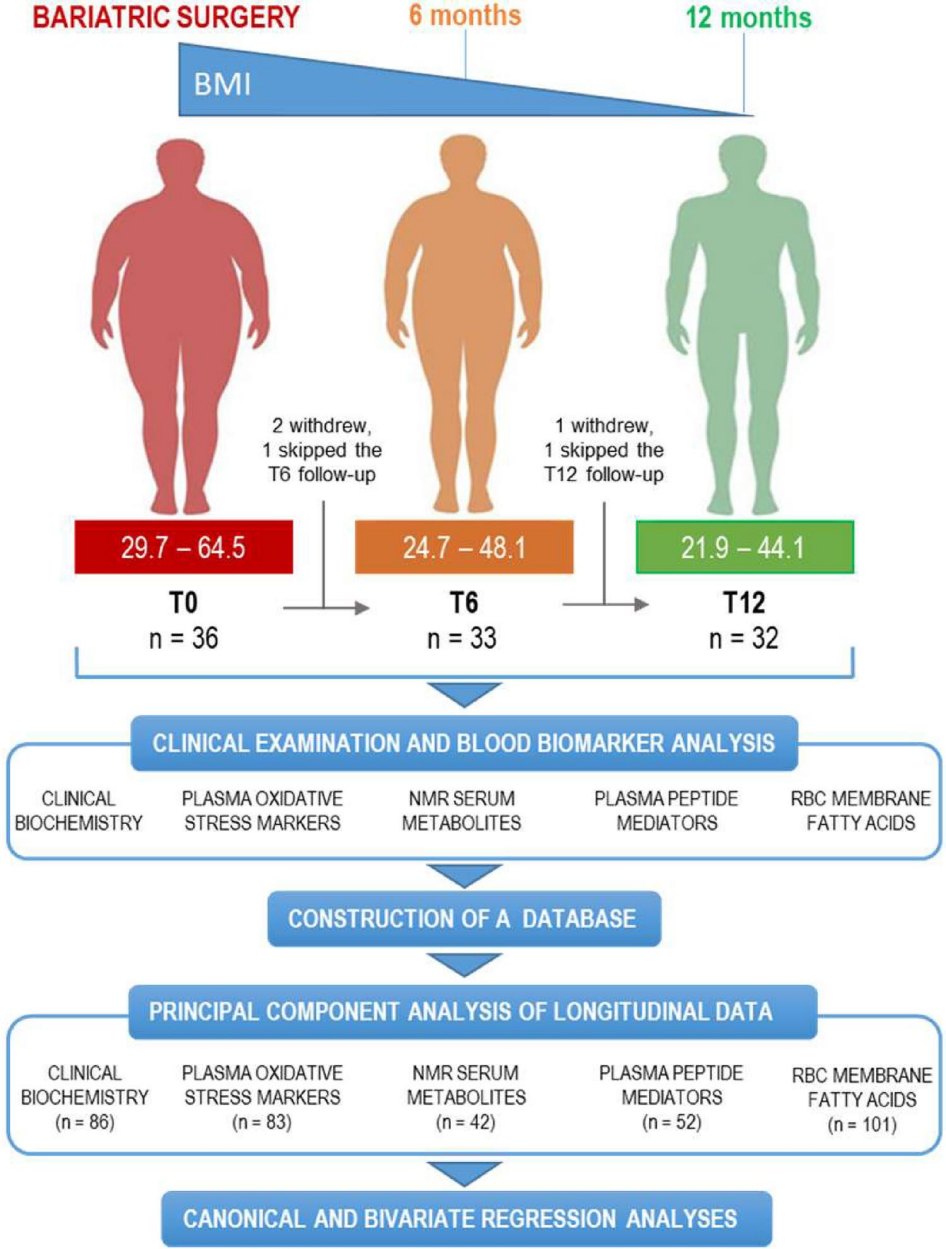
Flow diagram of the study. For each time of the study, the number of patients and the range (min–max) of patients’ BMI are displayed. The number of observations included in PCA analysis for each biomarker dataset is indicated in parentheses.

For this longitudinal study thirty-six subjects affected by severe obesity were recruited and subjected to BS (86% purely restrictive). Thirty-two patients (89%) completed the study. The study design is presented in Fig. 1 and the participant characteristics are shown in Table 1. A hundred and five variables including clinical and clinical biochemistry parameters, oxidative stress and metabolic markers, peptide mediators and RBC membrane lipids were analyzed at baseline (pre-surgery, T0), 6 (T6) and 12 (T12) months post-surgery. As expected, a significant weight loss was observed at 6 and 12 months after BS (− 28.6 ± 9.8 kg corresponding to − 24 ± 6% and − 36.6 ± 14 kg corresponding to − 31 ± 9%, respectively; mean ± SD). Full results, as well as a synthetic description of the temporal trends of selected blood biomarkers, are presented in supplementary material (Supplementary Table S1 and Supplementary Fig. S1).

**Table 1:** Patients’ characteristics.

	T0 (Baseline)	T6 (6 months)	T12 (12 months)
N subjects	36	33	32
Male/Female (n)	3/33	2/31	2/30
Age (years)	46.9 (38.9–57)	47.9 (41.7–58.5)	48.7 (42.5–59)
BMI (kg/m^2^)	44.3 (39.7–48.3)	33.5 (30.6–34.8)	29.8 (27.1–32.2)
Waist circumference (cm)	122 (114–129)	100 (90–107)	93 (84–102)
Hip circumference (cm)	141 (132–150)	120 (110–127)	111 (104–119)
Hypertension (n)	15 (42%)	12 (36%)	10 (31%)
Dyslipidemia (n)	22 (61%)	14 (42%)	9 (28%)
Impaired fasting glucose (n)	9 (25%)	1 (3%)	2 (6%)
Metabolic syndrome (n)	19 (53%)	8 (24%)	5 (16%)
Male/female (n)	3/16	2/6	1/4
Age (years)	52.1 (44.4–58.7)	54.4 (45.4–60)	59.1 (56.7–60.4)

Continue variables are expressed as median (IQ range). Discrete variables are reported as number of subjects (%).

**Figure 2:**
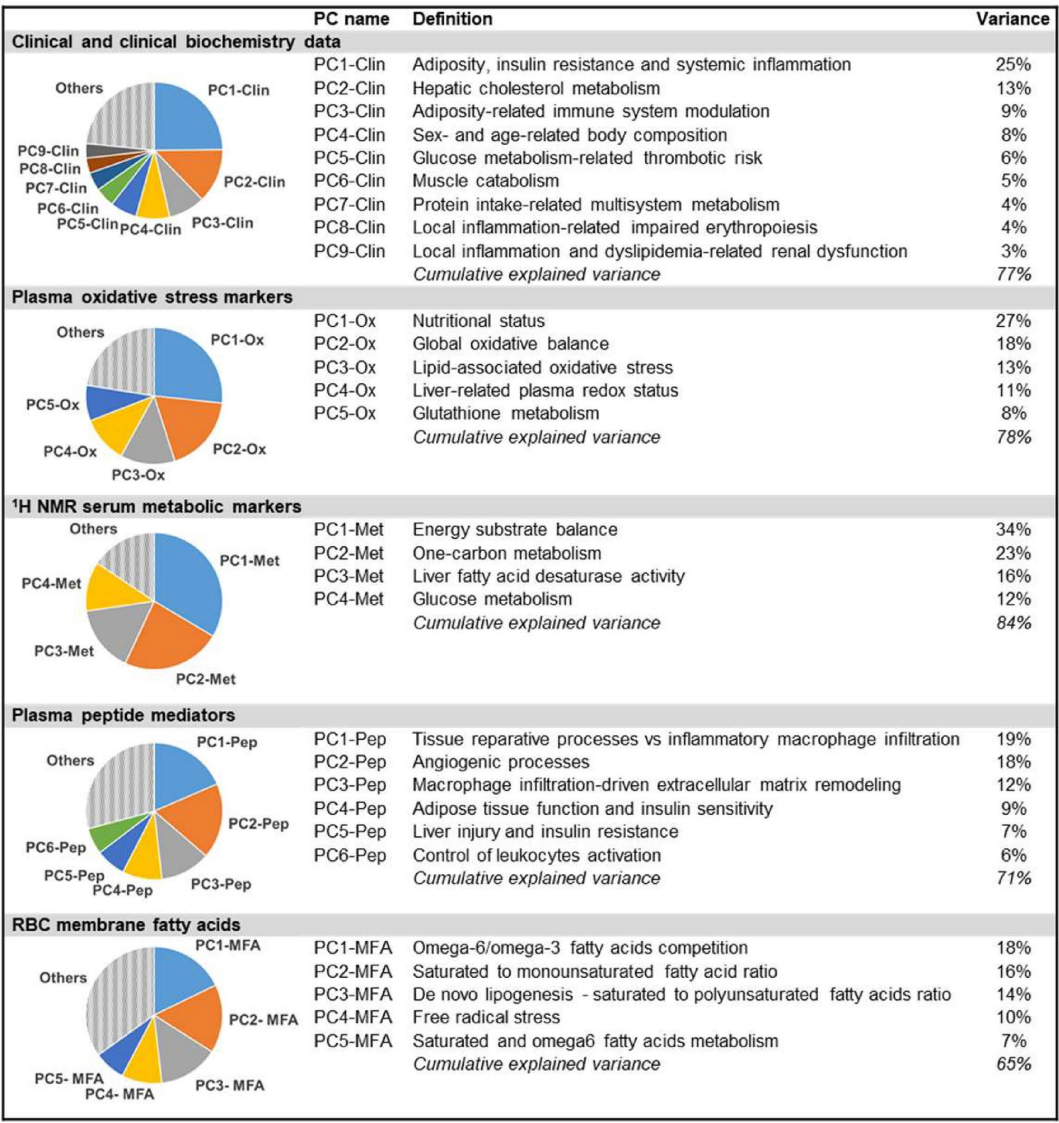
Principal components analysis. The main PCs extracted from each data set are presented in terms of percentage of explained variance and biological significance as inferred by component loading matrix (see Supplemental material for details).

By applying PCA, the PCs of each data set were extracted and, based on the component loading matrix (Supplementary Table S2), their biological meaning was decoded as presented in Fig. 2 (details about PCs interpretation are provided in Supplementary material and Supplementary Table S3).

**Figure 3:**
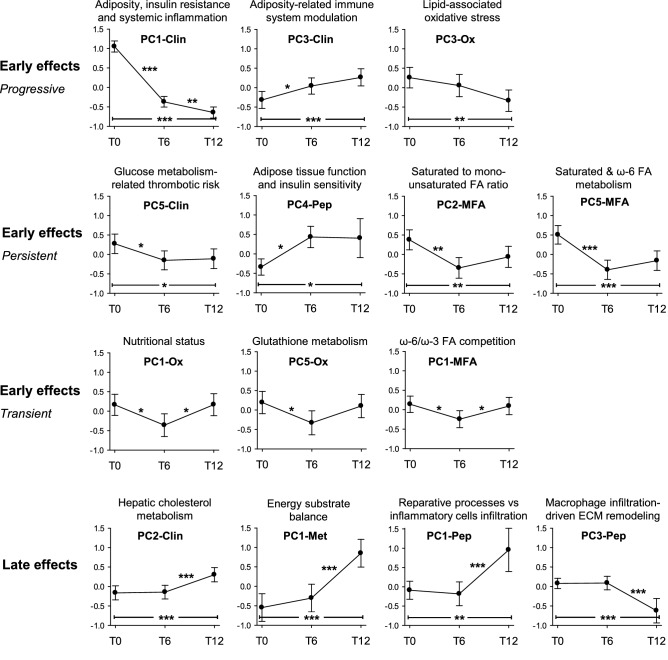
Early and late effects of bariatric surgery as assessed by PCs temporal trends. PC values at baseline (T0), six (T6) and twelve (T12) months from BS are reported. Values are means with 95% CI. *p ≤ 0.05; **p ≤ 0.01; ***p ≤ 0.001, repeated measures ANOVA with contrasts. *ECM* extracellular matrix.

The BS represents a relevant perturbation of the entire organism equilibrium state. The consequences of this abrupt change of the system equilibrium were monitored by comparing PC scores at the three time points of the study (Fig. 3).

In the 12 months after BS, PC1-Clin, PC3-Clin and PC3-Ox scores showed a continuous, progressive change (Fig. 3, first series), indicating an early and long-lasting effect of BS consisting in the reduction of adiposity, insulin resistance and systemic inflammation, in the modulation of the immune response as well as in the abatement of lipid-associated oxidative stress. Accordingly, after 6 months from BS the thrombotic risk related to glucose metabolism (PC5-Clin) was significantly reduced and the adipose tissue function (PC4-Pep) was significantly increased, two effects which persisted up to 1 year (Fig. 3, second series, first two plots). In the same time frame, BS induced the remodeling of the cell membrane composition as shown by the significant decrease of the SFA to MUFA ratio (PC2-MFA) and the activation of SFA and omega-6 FA metabolism (PC5-MFA) (Fig. 3, second series, last two plots). Nutritional status (PC1-Ox), glutathione metabolism (PC5-Ox) and omega-6/omega-3 FA ratio (PC1-MFA) showed a transient reduction at 6 months after surgery, an effect probably linked to the diet restrictions occurring in the first months post-BS (Fig. 3, third series).

Some effects became evident only late after surgery (Fig. 3, fourth series). PC2-Clin increased at T12 indicating a tardive unbalance of the hepatic metabolism toward cholesterol metabolism. In the same time-frame, an unbalance of the energetic metabolism (PC1-Met), together with a shift in adipose tissue remodeling towards a less inflamed phenotype (PC1- and PC3-Pep) were observed. Other PCs did not show significant changes after surgery (Supplementary Fig. S2).

### Oxidative stress is the most predictive feature of clinical outcome

**Figure 4:**
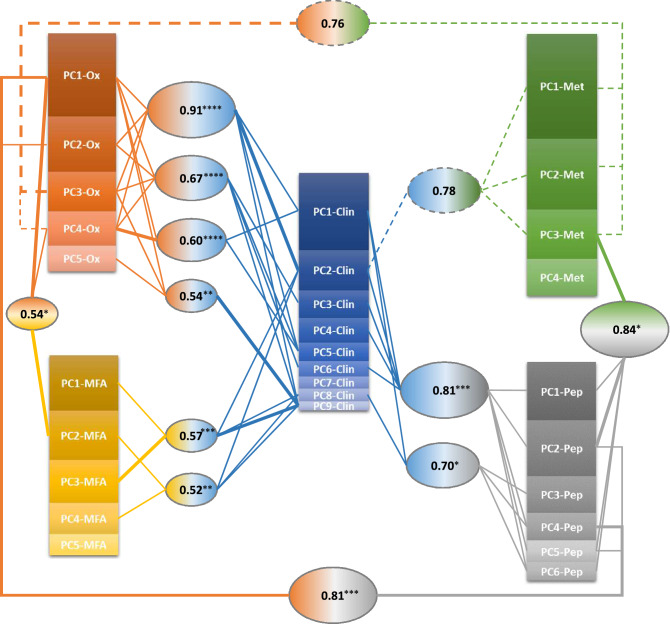
Graphical representation of the canonical correlation analysis. Each oval box represents a significant correlation (r) between linear combinations of PCs from two data set, with lines indicating the PCs from each data set significantly contributing to the combination. Thick lines indicate prominent contributions, i.e. the PC with a coefficient at least 1.5 times greater in modulus than that of any other PC in the same combination. PC box size is proportional to the variance explained by that PC within the dataset. ****p < 0.0001; ***0.0001 ≤ p < 0.001; **0.001 ≤ p < 0.01; *0.01 ≤ p < 0.05. Dashed lines indicate not statistically significant (0.05 < p < 0.15) though very strong (r > 0.7) correlations.

Canonical correlation analysis^32^ was used to estimate the mutual connections among the different physiological domains, represented in terms of their principal component scores. The topology of the interaction network generated by this analysis is represented in Fig. 4.

The clinical profile emerged as the hub of the correlation network as expected by the fact that it is the shared ’end-point’ of the different biological processes analyzed.

At a finer level of detail, the following observations can be made. First, it is the lipid metabolism and in particular cholesterol metabolism (represented by both PC2-Clin and PC9-Clin) and not the carbohydrate metabolism or weight loss (represented by the PC1-Clin) that has greater interactions with the other metabolic networks. The nutritional status and oxidative stress play a relevant role in many and different metabolic networks. The strongest correlation (r = 0.91, p < 0.0001) is between a combination of the components PC1-Clin/PC2-Clin/PC9-Clin/PC5-Clin, which express the main dysmetabolisms (carbohydrate and lipid), and a combination of the first four components of the oxidative stress markers dataset, including nutritional status, oxidative balance and oxidative damage biomarkers. At difference, RBC membrane fatty acids dataset shows significant connections only with nutritional status (PC1-Ox; r = 0.54, p = 0.010) and cholesterol metabolism and related disorders (PC2-Clin and PC9-Clin; r = 0.57, p < 0.001 and r = 0.52, p < 0.01).

### The functional significance of PC1-Clin and PC2-Clin interaction network

The bivariate correlations of PC1-Clin and PC2-Clin with each PC of the other data sets were then analyzed to gain insights into the functional significance of the interaction network topology (Table 2). PC1-Clin and PC2-Clin were selected because of their significance in terms of explained variance within the clinical dataset, and of their high number of connections in the canonical analysis.

**Table 2:** Bivariate correlation analysis of PC1-Clin and PC2-Clin versus the PCs of the other datasets.

		PC1-Clin Adiposity, insulin resistance and systemic inflammation			PC2-Clin Hepatic cholesterol metabolism		
		*r*(*s*)	n	p	*r*(*s*)	n	p
**Plasma oxidative stress markers**							
PC1-Ox	Nutritional status	*–*	–	–	0.471	71	< 0.001
PC2-Ox	Global oxidative balance	− 0.453	71	< 0.001	− 0.263	71	0.027
PC3-Ox	Lipid-associated oxidative stress	0.328	71	0.005	0.293	71	0.013
PC4-Ox	Liver-related plasma redox status	*–*	–	–	0.281	71	0.018
PC5-Ox	Glutathione metabolism	*–*	–	–	− 0.236	71	0.048
^1^**H NMR serum metabolic markers**							
PC2-Met	One-carbon metabolism	*–*	–	–	− 0.502	33	0.003
PC4-Met	Glucose metabolism	0.584	33	< 0.001	*–*	–	–
**Plasma peptide mediators**							
PC1-Pep	Tissue reparative processes vs inflammatory macrophage infiltration	*–*	–	–	− 0.339	43	0.026
PC2-Pep	Angiogenic processes	*–*	–	–	0.467	43	0.002
PC4-Pep	Adipose tissue function and insulin sensitivity	− 0.462	43	0.002	*–*	–	–
**RBC membrane fatty acids**							
PC1-MFA	ω-6/ω-3 FA competition	*–*	–	–	− 0.337	86	0.002
PC4-MFA	Free radical stress	*–*	–	–	0.257	86	0.017
PC5-MFA	SFA and ω-6 FA metabolism	0.237	86	0.028	*–*	–	–

Only significant correlations are shown.

In agreement with the results of canonical analysis, PC2-Clin shared a higher number of significant correlations than PC1-Clin. PC1-Clin, i.e. adiposity, insulin resistance and systemic inflammation, was highly correlated to oxidative imbalance (PC2-Ox) and increased oxidative damage (PC3-Ox), as well as to impaired peripheral glucose metabolism (PC4-Met) and adipose tissue function (PC4-Pep). PC1-Clin also showed a significant, albeit weaker, correlation with decreased saturated and omega 6 fatty acids metabolism (PC5-MFA).

PC2-Clin, i.e. hepatic cholesterol metabolism, was strongly correlated with diet quantity and quality (nutritional status, PC1-Ox) and ω-6/ω-3 FA competition, PC1-MFA), disorders in one carbon metabolism (PC2-Met) and vascular remodeling (PC2-Pep). Less strong still multiple and consistent correlations were also found between PC2-Clin and oxidative stress related biochemical networks (PC2-Ox, PC3-Ox, PC4-Ox, PC5-Ox and PC4-MFA) as well as with tissue macrophage infiltration (PC1-Pep).

## Discussion

In this study, the dynamics of the clinical and metabolic profile of patients with severe obesity undergoing BS was followed up to 1 year post-surgery by using a comprehensive data-driven approach based on PC and correlation analyses. This is the first time an integrative analysis of biomarkers data belonging to five physiological domains (clinics, oxidative stress, metabolism, peptide mediators and cell membrane lipids) is performed.

This analysis showed that in the first 6 months after BS relevant clinical and metabolic improvements take place, but then the organism slowly reaches a new equilibrium with some metabolic and nutritional disturbances still persisting or even arising 1 year post-surgery. This scenario is captured by the first two PCs of the clinical dataset, PC1-Clin and PC2-Clin, whose dynamics efficiently sum up the good and the bad effects of BS, respectively. The PC1-Clin trend shows that anthropometric indices and insulin sensitivity rapidly improve mostly within 6 months after surgery and this phenomenon correlates to decreased oxidative stress and improved adipose tissue metabolic function and peripheral glucose metabolism. Our data support the notion that upon BS, peripheral (skeletal muscle and adipose tissue) insulin sensitivity improves in proportion to weight loss^33^ and both changes are inter-related with improvements of adipose tissue function^34^ and oxidative balance^35^.

By performing a post-surgery follow-up of FA in membrane phospholipids, we provide new evidence that insulin sensitivity and weight loss are also associated with increased activity of the membrane FA transforming enzymes elongase-6 and delta5-desaturase. An increase in the estimated activity of these enzymes in serum has been shown in BS patients with higher weight loss^36^ and a negative association with insulin resistance has been observed in both serum and muscle cell membranes^37,38^. Although our results do not allow to establish a cause-effect relationship, they show that BS promotes the recovery of FA transforming activities along with weight loss and recovery of insulin sensitivity. Concomitantly, a significant decrease of the RBC membrane SFA/MUFA ratio (PC2-MFA) unrelated to PC1-Clin decrease but strongly associated to the nutritional status (PC1-Ox) was observed. Subjects with obesity are characterized by a higher SFA/MUFA ratio than NW subjects (this study and^24,26^). Here we show that BS promotes the normalization of this ratio by a diet-dependent mechanism which is largely independent on weight-loss and glucose-metabolism improvements. Actually, the PCs’ interaction network clearly shows that main determinants of the cell membrane FA composition in bariatric patients are the nutritional status, on one hand, and the cholesterol metabolism and related disorders, on the other hand. The glucose metabolism-related disorders have indeed a relatively little impact on the global membrane FA balance.

The temporal changes of the second PC of the clinical dataset, PC2-Clin, shows a tardive increase of the hepatic lipoprotein cholesterol metabolism. It is believed that BS affects lipoprotein profiles through multiple mechanisms which involve weight loss dependent and independent mechanisms^39^. Our study identifies two main general mechanisms by which BS affects lipids levels. The first one, represented by PC1-Clin and its dynamics, associates the reduction of triglycerides and the increase of HDL levels to weight loss and recovery of insulin sensitivity. The second one, represented by PC2-Clin and its dynamics, is independent of weight loss and associates the increase of total and LDL cholesterol levels to worse hepatic functions. Different BS procedures impact non-HDL cholesterol differently, with greater benefits achieved following malabsorptive vs. restrictive procedures^40^. Therefore, since restrictive surgical procedures were mostly adopted in our study, the lack of a positive effect of BS on TChol and LDL is not totally unexpected. On the other hand, this evidence sounds a note of caution on the generalizability of our results to malabsorptive surgical procedure such as Roux-en-Y gastric bypass (RYGB). Although the underlying mechanisms remain to be elucidated, the correlation analysis suggests that PC2-Clin increase is associated to other potentially detrimental effects. In fact, PC2-Clin was positively correlated to oxidative stress, tissue macrophage infiltration, angiogenic processes and defective one-carbon metabolism. These correlations are not surprising since oxidative susceptibility of plasma lipoproteins may account for increased oxidative damage^41^ and cholesterol is known to promote adipose tissue macrophage accumulation^42^ and regulate angiogenesis^43^. A negative association between one-carbon metabolism markers and circulating non-HDL lipoproteins has been already reported in overweight people, though the underlying mechanism are unclear^44^.

Network correlation analysis of biology of disease^32^ has demonstrated that under stress the connectivity among correlated biological traits may increase allowing to identify clusters of biological attributes of the system. In our study, the adoption of this analysis shows that, under stress (i.e. BS), the different biological traits identified (i.e. the PCs) are highly connected allowing macroscopic properties to emerge. The clinical field emerged as the hub of our network, with the cholesterol metabolism sharing the highest number of connections (Fig. 4). Among the other fields, the oxidative stress is the only one that shared connections with every other field, thus emerging as the most predictive feature at molecular level of clinical alterations in people with severe obesity. It is known that oxidative stress promotes obesity^45–48^ and that, in turn, adiposity leads to increased oxidative stress^49,50^, thus leaving open the question on which comes first^51^. Our findings highlight the central role of oxidative stress in many different biological networks affected by obesity and indicate oxidative stress biomarkers not only as a hallmark of the severe obesity state but also as useful tools to monitor the health state of BS patients.

The second important message coming out from the correlation network is that, among the clinical PCs, PC2 more than PC1 is correlated with the other fields. This indicates that the change in cholesterol metabolism induced by BS relies on a more complex metabolic network than those related to weight loss and glucose metabolism. It has been recently questioned whether weight loss is the best predictor of the success of BS. Indeed, in a recent study, no correlation was found between improvements in cardiovascular risk factors and surgery-induced weight loss^52^. Our study demonstrates that, following BS, cholesterol metabolism behaves in a weight loss-independent way, which means that BS-induced weight loss did not convey information on this important part of the lipid metabolism. Moreover, by using weight loss, post-surgery perturbances of other important physiological networks related to cholesterol metabolism would be neglected as well.

BS is known to lead to durable weight loss and reduced risk of cardiovascular disease, type 2 diabetes and cancer^53^. Our study confirms that BS has a strong impact on a variety of clinical and metabolic parameters leading to a better health profile. However, BS is not a cure. In fact, some disturbances in cholesterol metabolism associated to pro-inflammatory and pro-oxidative processes as well as to metabolic alterations may emerge 1 year after surgery. In addition, signs of nutrient deficit, as shown by alterations in membrane lipidomic profile, persist after surgery. These unresolved aspects could partially explain why, even if BS is associated with longer life expectancy than usual obesity care, overall mortality (mostly due to cardiovascular diseases and cancer) among patients who had undergone BS remains higher than in the general population^54,55^.

Our study has two main strengths. First, the wide array of biomarkers analyzed which allowed to highlight the dynamical interactions of many different biological processes upon surgery. Second, the unsupervised statistical approach adopted which allowed to both summarize the information and shed light on the underlying relationships inherent to the original dataset. Our study also presents limitations. The number of patients analyzed is relatively small and for this reason weaker associations could have been missed. However, the longitudinal design of the study, by using repeated observations at individual level, can provide high accuracy when observing changes. To this regard, it should be taken into account that this study had a main explorative nature aimed to detect interesting signals that may be worth of further investigation in subsequent better-powered studies. In addition, since we did not adopt specific, quantitative measures of compliance with diet, the possibility of residual confounding factors could not be ruled out. It should also be emphasized that most of our patients were women and that they underwent mostly restrictive BS, therefore we do not know whether the same changes would be observed in a male cohort of patients or upon malabsorptive or mixed surgical procedures.

Overall, our study shows the power of multi-level correlation analysis to uncover the network of physiological changes induced by BS. Importantly, it allowed to identify a constellation of weight-independent disturbances which are disregarded with the current practice to use weight loss as surrogate of BS success. This approach has a strong potential in identifying the most predictive biomarkers, hence it could be successfully employed to improve the post-surgery monitoring and management of bariatric patients.

## Supplementary Information

Supplementary Information.

## Data Availability

The datasets generated during the current study are available from the corresponding authors upon reasonable request.
