# Supplementary material for: A multi-marker integrative analysis reveals benefits and risks of bariatric surgery
Source: Sci Rep. 2022 Nov 7;12:18877. doi: 10.1038/s41598-022-23241-6 (PMC9640526; doi:10.1038/s41598-022-23241-6)
Supplement: Supplementary file 1 — Supplementary Information. [file 41598_2022_23241_MOESM1_ESM.pdf]

Supplementary material to

A MULTI-MARKER INTEGRATIVE ANALYSIS REVEALS BENEFITS AND RISKS OF BARIATRIC SURGERY

Simonetta Palleschi[1,10,§], Valeria Guglielmi[2,3,10], Lorenza Nisticò[4,10], Carla Ferreri[5], Claudio Tabolacci[6], Francesco Facchiano[6], Egidio Iorio[7], Alessandro Giuliani[1], Sonia Brescianini[4], Emanuela Medda[4], Corrado Fagnani[4], Barbara Rossi[1], Anna Minoprio[1], Mattea Chirico[7], Maria Elena Pisanu[7], Federica Di Nolfo[8], Paola Fortini[1], Valeria Simonelli[1], Sara Baccarini[6], Serena Laterza[2,3], Tiziana Morretti[2,3], Ambra Dell'Orso[1], Federico Manganello[1], Paolo Gentileschi[9], Paolo Sbraccia[2,3] & Eugenia Dogliotti[1,§]

[1] Department of Environment and Health, Istituto Superiore di Sanità, 00161 Rome, Italy
[2] Internal Medicine Unit and Obesity Center, University Hospital Policlinico Tor Vergata, 00133 Rome, Italy
[3] Department of Systems Medicine, University of Rome Tor Vergata, 00133 Rome, Italy
[4] Center for Behavioral Sciences and Mental Health. Istituto Superiore di Sanità, 00161 Rome, Italy
[5] Consiglio Nazionale delle Ricerche, ISOF, 40129 Bologna, Italy
[6] Department of Oncology and Molecular Medicine, Istituto Superiore di Sanità, 00161 Rome, Italy
[7] Core Facilities, Istituto Superiore di Sanità, 00161 Rome, Italy
[8] Lipidomic Laboratory, Lipinutragen srl, 40128 Bologna, Italy
[9] Bariatric Surgery Unit, Department of Surgery, University of Rome Tor Vergata, 00133 Rome, Italy

[10] These authors contributed equally: Simonetta Palleschi, Valeria Guglielmi and Lorenza Nisticò
[§] Corresponding authors: simonetta.palleschi@iss.it; eugenia.dogliotti@iss.it.

This file includes:

Principal Component analysis (PCA) and PC interpretation methods …………………………….. 2
Synthetic description of the temporal trends of selected blood biomarkers after BS………………. 3
Figure S1. Effects of bariatric surgery on selected blood biomarkers……………………………… 4
Table S1. Biomarkers values before and after surgery……………………………………………... 5
Table S2. Principal component analysis: loading matrices of the main PCs extracted from each data set…………………………………………………………………………………………....... 10
Table S3. Biological meaning of the main PCs extracted from each data set, as inferred by the maximally loaded variables………………………………………………………………………… 13
Figure S2. Temporal trends of PCs that are not significantly affected by bariatric surgery………... 15
References………………………………………………………………………………………… 16

Principal component analysis (PCA) and PC interpretation methods

In PCA, for each local data set (clinical and clinical biochemistry data, plasma oxidative stress markers, ^{1}H NMR-based serum metabolic markers, plasma peptide mediators and RBC membrane fatty acids), the raw data matrix had as rows (statistical units) the individual samples at each time point and as variables the experimental measurements.

Some data were missing due to incomplete follow-up (11% at T12) or technical failures (lost or not available samples, especially for peptide mediators assays). N values for each variable and each time point are provided in Supplementary Table S1. As for 1H-NMR serum metabolic markers, a subset of patients (14) has been studied and these have complete data for all time points. All cases (i.e., patients) - including those with missing data at follow-up - were used in the analysis, assuming that observations were lost to follow up under a MCAR (Missing Completely at Random) mechanism. For each time of the study, only cases with a complete set of data were entered in the local PCA. Like any multivariate spectral analysis, PCA asks for a full rank data matrix with no missing observation. While it is possible to use 'imputation procedures' to fill the 'gaps', we preferred not to insert a possible bias in the analysis. Consequently, the variables of the peptide mediators data set with too many gaps were not included into the local PCA that, in any case, maintained a sufficient number of variables to allow (by loading inspection) assigning a biological meaning to extracted components.[1] As for the the Clinical and clinical biochemistry dataset, the variables of the single white blood cell fractions (lymphocytes, monocytes, granulocytes, eosinophils and basophils) were entered into the local PCA instead of the global variable "White blood cells". For each local PCA, the number of extracted components was determined by the Cattell scree test that takes into consideration the progressive decay of variance explained by higher order components; hence, the components identified as *bona fide* signals were selected upon elimination of the noise-dominated ones (i.e. low eigenvalues).[2]

The biological meaning of PCs was inferred by the component loading matrix (Supplementary Table S2). To provide an example, in the clinical data set a 9 component solution accounts for 77% of total variance. In the case of PC1-Clin (25% of total variance), the maximally loaded variables - WC, insulin, HOMA-IR, weight, BMI and hsCRP - all show a positive coefficient and are related to the insulin resistance and systemic inflammation associated to central obesity. Therefore, this component has been interpreted as "Adiposity, insulin resistance and systemic inflammation". By following the same reasoning based on the loading pattern, the PCs of all data set have been decoded as presented in Supplementary Table S3.

Synthetic description of the temporal trends of selected blood biomarkers after BS

In general, the clinical and metabolic profile of patients recovered quickly post-surgery but with some notable exceptions. The most significant changes are displayed in Figure S1 and full results are presented in Table S1. Although an early improvement in anthropometric parameters (waist circumference, WC, from 122 cm at T0 to 100 cm at T6, median values, $p<0.001$, Wilcoxon signed-rank test), glucose metabolism (homeostatic model assessment for insulin resistance, HOMA-IR, from 4.32 at T0 to 1.51 at T6, $p<0.001$) and systemic inflammation (C-reactive protein, CRP, from 7.64 mg/l at T0 to 2.4 mg/l at T6, $p<0.001$), Low Density Lipoprotein (LDL) levels did not vary (124 mg/dl at T0), and total cholesterol (TChol) showed a modest, tardive increase (from 195 mg/dl at T6 to 202 mg/dl at T12, p= 0.044), as already described after purely restrictive interventions like sleeve gastrectomy.[5,6] Advanced Oxidation Protein Products (AOPP, a marker of plasma proteins oxidation) decreased early after BS (from 48 µmol/l at T0 to 40 µmol/l at T6, p=0.02). At difference, protein-normalized total free thiols (SHp, a marker of plasma redox status) and malondialdehyde (MDA, a marker of lipid peroxidation) did not improve significantly up to one year from surgery. A temporal dissociation was observed for markers of adipose tissue function, with early changes in the circulating levels of leptin (from 87 ng/ml at T0 to 27 ng/ml at T6, $p < 0.001$) and adiponectin (from 3.7 ng/ml at T0 to 5.7 ng/ml at T6, $p<0.001$) but later decrease of monocyte chemoattractant protein-1 (MCP-1; from 309 pg/ml at T0 to 219 at T12, $p<0.001$) that plays a critical role in adipose tissue macrophage recruitment as well as in the development and progression of obesity-related metabolic disease.[7] The plasmatic levels of both interleukin-6 (IL-6) and tumor necrosis factor-α (TNF-α) decreased in the post-surgery period (though the changes were significant only when these biomarkers were assayed with the Luminex platform: IL-6 went from 2.1 pg/ml at T0 to 1.0 pg/ml at T12, p=0.028, and in the same period TNF-α went from 9.0 pg/ml to 7.7 pg/ml, p = 0.005), confirming that BS efficiently reduces the inflammatory status of treated subjects.

Despite the normalization of glucose levels, lactate levels which were also higher than in NW subjects (34.9‰ in bariatric patients at T0 vs 25.8‰ in NW subjects, median values, p=0.01, Mann-Whitney test, ^{1}H NMR spectroscopy data expressed in ‰ metabolite/all metabolites), did not change after BS, and alanine levels showed a late decrease (from 46.7‰ at T0 to 35.2‰ at T12, p=0.001), thus suggesting that some alteration in redox and energetic metabolism still persisted until one year after surgery. Serum lipoproteins and fatty acids, as assessed by the L1 and L3 ^{1}H NMR signal, significantly increased after BS (from 207‰ at T0 to 231‰ at T12, p = 0.005 and from 152‰ at T0 to 162‰ at T12, p = 0.011, respectively) confirming the trend of total cholesterol.

BS partially restored RBC membrane fatty acid (FA) composition by significantly reducing the excess of palmitic acid, an indicator of de novo lipogenesis (from 22.9% at T0 to 22.3% at T12, p = 0.009, data expressed as % fatty acid /all fatty acids), and omega-6 dihomo-γ-linolenic acid (DGLA; from 2.4% at T0 to 1.9% at T12, $p<0.001$). The level of oleic acid increased after the bariatric intervention (from 17.4% at T0 to 18.2% at T12, $p<0.001$) promoting a shift in the quality of membrane lipids toward MUFA together with a progressive decrease of the SFA/MUFA ratio (from 2.06 at T0 to 1.99 at T12, p = 0.021). However, the deficiency of RBC membrane linoleic acid and, more in general, of polyunsaturated FA (PUFA: 39.6% in bariatric patients at T0 vs 41.2% in NW subjects, p = 0.002) was not recovered while an early increase of the pro-inflammatory arachidonic acid (AA) lasting up to one year from surgery was observed (from 18.5% at T0 to 19.1% at T12, p = 0.032), highlighting some unresolved issues in lipid intake and transformation which potentially makes the patient more prone to inflammation.

Figure S1. Effects of bariatric surgery on selected blood biomarkers. Patients values at baseline (T0), six (T6) and twelve (T12) months from BS are reported; normal-weight (NW) subjects values are shown as reference. Boxes indicate median and IQR. Whiskers indicate the 90th and 10th percentiles. All outliers are showed. WC = waist circumference; HOMA-IR = Homeostatic model assessment for insulin resistance; CRP = C-reactive protein; LDL = LDL cholesterol; tCHOL = total cholesterol; AOPP = Advanced Oxidation Protein Products; SHp = total free thiols normalized on total proteins; MDA = malondialdehyde; MCP-1 =Monocyte Chemoattractant Protein-1; DGLA = Dihomo-γ-linolenic acid; PUFA = Total Polyunsaturated fatty acids. a = ‰ metabolite/all 1H NMR serum metabolites. b = % fatty acid/all membrane fatty acids. * = p ≤ 0.05; ** = p ≤ 0.01; *** = p ≤ 0.001. The complete dataset of results is reported in Table S1.

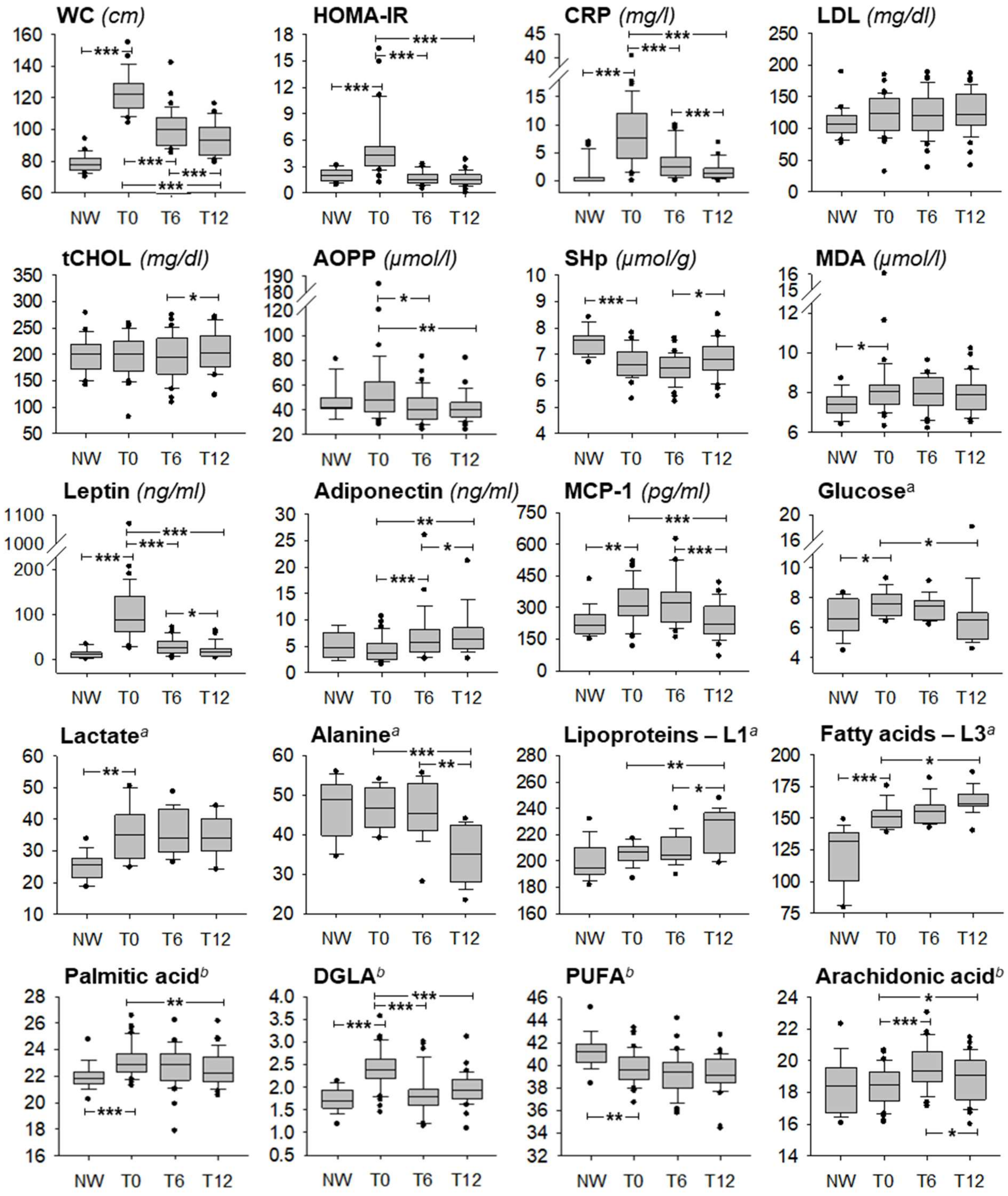

Table S1. Biomarkers levels before and after surgery. Median values and interquartile ranges are reported for the enrolled patients (OB) before (T0) and six months (T6) and twelve months (T12) after bariatric surgery. Values from a small cohort of normal weight (NW) subjects are also reported as reference.

	NW subjects	OB at T0	OB at T6	OB at T12	OB at T0 vs NW	OB T0 vs T6	OB T6 vs T12	OB T0 vs T12
	n *Median* *(IQ range)*	*n* *Median* *(IQ range)*	*n* *Median* *(IQ range)*	*n* *Median* *(IQ range)*	*p-value**	*p-value***	*p-value***	*p-value***
**Clinical and clinical biochemistry markers**								
**Weight (Kg)**	16 67.15 (60.65-68.95)	36 115.5 (103-130.5	33 86.7 (77.3-96)	32 76.5 (68.05-91.6)	<0.001	<0.001	<0.001	<0.001
**Height (cm)**	16 165.5 (160.8-169.5)	36 162.5 157.5-167.5	33 162 158-168	32 160 157.5-166.5				
**Body mass index (BMI, Kg/m^2)**	16 23.52 (22.59-24.58)	36 44.28 (39.74-48.32)	33 33.45 (30.60-34.80)	32 29.81 (27.10-32.21)	<0.001	<0.001	<0.001	<0.001
**Waist Circumference (WC, cm)**	16 78 (74.5-82)	33 122 (114-129)	33 100 (90-107)	32 93.25 (83.75-101.5)	<0.001	<0.001	<0.001	<0.001
**Hip Circumference (HC, cm)**	15 97.5 (95-101.7)	33 141 (132-150)	33 120 (110-127)	32 110.5 (103.75-119)	<0.001	<0.001	<0.001	<0.001
**Waist-Hip ratio**	15 0.81 (0.75-0.86)	33 0.86 (0.83-0.91)	33 0.83 (0.80-0.89)	32 0.83 (0.79-0.88)	0.020	0.032		0.006
**Glycemia (mg/dl)**	16 82 (78-89.5)	36 93 (87-102.5)	33 81 (77-87)	32 85 (80-91.5)	0.002	<0.001		<0.001
**Insulin (µU/ml)**	16 9.69 (6.38-11.69)	36 17.95 (14.78-22.13)	33 7.8 (6.1-8.7)	31 7.4 (5.4-9.6)	<0.001	<0.001		<0.001
**Homeostatic model assessment for insulin resistance (HOMA-IR)**	16 1.99 (1.34-2.59)	36 4.32 (3.00-5.21)	33 1.51 (1.17-1.98)	32 1.50 (1.05-2.07)	<0.001	<0.001		<0.001
**HbA1c (mmol/mol)**	15 33 (32-34)	35 38 (36-43)	33 34 (32-37)	32 34 (32-36)	<0.001	<0.001		<0.001
**Uric acid (mg/dl)**	16 3.55 (2.75-4.45)	31 5.3 (3.8-6.1)	33 4.7 (3.9-5.2)	32 3.9 (3.45-4.9)	0.002	0.036	0.002	<0.001
**Creatinine (mg/dl)**	16 0.74 (0.67-0.88)	36 0.67 (0.57-0.77)	32 0.62 (0.57-0.71)	31 0.64 (0.57-0.7)	0.046			
**Blood urea nitrogen (mg/dl)**	16 27 (23.5-35.5)	36 30.5 (24.5-35)	30 26 (22-31)	32 29 (24-36)		0.003	0.008	
**Red Blood Cells (RBC, 10^6/µl)**	16 4.82 (4.66-4.98)	36 4.97 (4.69-5.17)	33 4.92 (4.59-5.15)	32 4.80 (4.61-5.19)				
**Platelets (10^3/µl)**	16 253 (218.5-268)	36 247 (235.5-311.5)	33 245 (220-312)	32 248.5 (218-314.5)				0.018
**White blood cells^ (WBC, 10^3/µl)**	16 6.19 (4.49-8.4)	36 7.69 (6.86-8.85)	33 7.19 (5.81-7.95)	32 6.63 (5.43-7.44)	0.037	0.001	0.007	<0.001
**Lymphocytes (10^3/µl)**	16 1.81 (1.29-1.93)	36 2.09 (1.83-2.30)	33 1.95 (1.75-2.37)	32 1.95 (1.62-2.35)	0.005			
**Monocytes (10^3/µl)**	16 0.40 (0.28-.048)	36 0.50 (0.40-0.58)	33 0.41 (0.36-0.5)	32 0.38 (0.32-0.46)	0.003	<0.001	0.016	<0.001
**Eosinophils (10^3/µl)**	16 0.08 (0.04-0.15)	36 0.15 (0.1-0.21)	33 0.11 (0.08-0.14)	32 0.11 (0.07-0.16)	0.047	0.025		0.005
**Basophils (10^3/µl)**	16 0.04 (0.03-0.06)	36 0.04 (0.03-0.04)	33 0.04 (0.03-0.05)	32 0.04 (0.03-0.05)				

	NW subjects	OB at T0	OB at T6	OB at T12	OB at T0 vs NW	OB T0 vs T6	OB T6 vs T12	OB T0 vs T12
	n<br>*Median*<br>*(IQ range)*	*n*<br>*Median*<br>*(IQ range)*	*n*<br>*Median*<br>*(IQ range)*	*n*<br>*Median*<br>*(IQ range)*	*p-value**	*p-value***	*p-value***	*p-value***
**Neutrophils ($10^3/\mu l$)**	16<br>3.27<br>(2.54-6.07)	36<br>4.66<br>(3.93-6.15)	33<br>4.49<br>(3.58-5.12)	32<br>3.91<br>(2.96-4.67)		0.006	0.003	<0.001
**Total Cholesterol (TChol, mg/dl)**	16<br>200<br>(173-218)	36<br>200.5<br>(168.5-224.5)	33<br>195<br>(163-230)	32<br>202<br>(176.5-234.5)			0.044	
**HDL Cholesterol (HDL, mg/dl)**	16<br>70.5<br>(53.5-90.5)	35<br>50<br>(41-57)	33<br>55<br>(47-62)	32<br>55.5<br>(49.5-67)	0.001	0.029	0.002	0.001
**Triglycerides (mg/dl)**	16<br>65<br>(56-94)	36<br>124<br>(96-179)	33<br>95<br>(73-126)	32<br>84.5<br>(75-110.5)	<0.001	<0.001		<0.001
**LDL Cholesterol (LDL, mg/dl)**	16<br>107.5<br>(92.8-120.6)	36<br>123.6<br>(95.8-148)	33<br>120.4<br>(96.8-147.2)	32<br>122.6<br>(105.3-154.8)				
**Aspartate Aminotransferase (AST, IU/L)**	16<br>15<br>(12-19.5)	36<br>20<br>(16-24)	33<br>18<br>(16-21)	32<br>20<br>(15-24.5)	0.019			
**Alanine transaminase (ALT, IU/L)**	16<br>23<br>(19-27)	36<br>29<br>(22.5-38.5)	33<br>18<br>(14-22)	32<br>18<br>(13-21.5)	0.034	<0.001		<0.001
**γ-Glutamyl Transferase (γGT, IU/L)**	16<br>18<br>(15-22.5)	33<br>25<br>(20-36)	33<br>16<br>(11-20)	32<br>13.5<br>(9-17.5)	0.013	<0.001	0.009	<0.001
**Alkaline Phosphatase (ALP, U/L)**	15<br>68<br>(59-75)	36<br>85<br>(72-98)	33<br>83<br>(71-97)	32<br>82<br>(63.5-98)	0.001			
**C-reactive protein, high sensitivity (hsCRP, mg/l)**	16<br>0.05<br>(0-0.63)	32<br>7.64<br>(4.03-12.05)	33<br>2.4<br>(0.9-3.9)	31<br>1.4<br>(0.6-2.2)	<0.001	<0.001	<0.001	<0.001
**TNF-α (pg/ml)**	16<br>4.2<br>(3.20-5.93)	36<br>6.6<br>(3.9-10.9)	33<br>7<br>(3.8-9.9)	32<br>6.65<br>(5.18-8.75)				
**IL-6 (pg/ml)**	16<br>7.86<br>(1.65-13.95)	35<br>15<br>(0.3-34.2)	32<br>0.75<br>(0.34-32.6)	32<br>0.82<br>(0.41-19.3)				
**Plasma oxidative stress markers**								
**Advanced Oxidation Protein Products (AOPP, µmol/l)**	14<br>41.5<br>(41-50)	33<br>48<br>(39-63)	31<br>40<br>(32-50)	31<br>40<br>(34-46)		0.020		0.007
**Total free thiols (SH, µmol/l)**	14<br>534.5<br>(502-578)	33<br>462<br>(434-502)	31<br>449<br>(426-485)	31<br>492<br>(444-531)	<0.001		0.043	
**Total proteins (g/dl)**	14<br>7.25<br>(6.8-7.6)	34<br>7<br>(6.8-7.3)	31<br>7<br>(6.6-7.3)	31<br>7.1<br>(6.5-7.6)				
**SH normalized on total proteins (SHp, µmol/g)**	14<br>7.55<br>(7-7.7)	33<br>6.6<br>(6.2-7.1)	31<br>6.5<br>(6.1-6.9)	31<br>6.8<br>(6.4-7.3)	<0.001		0.012	
**Malondialdehyde (MDA, µmol/l)**	13<br>7.4<br>(7-7.7)	32<br>8.05<br>(7.4-8.4)	27<br>8<br>(7.4-8.8)	27<br>7.9<br>(7.1-8.4)	0.049			
**Vitamin A (VitA, µmol/l)**	11<br>1.9<br>(1.5-2.1)	33<br>1.39<br>(1.25-1.76)	31<br>1.3<br>(1-1.62)	31<br>1.55<br>(1.39-1.96)	0.006	0.026	0.006	
**Vitamin E (VitE, µmol/l)**	11<br>25<br>(23-30.6)	33<br>33<br>(29-37)	31<br>30<br>(25-34)	31<br>30<br>(26-35)	0.018	0.014		
**Lipid-standardized[a] Vitamin A (VitAnorm, µmol/l)**	11<br>1.9<br>(1.79-2.05)	33<br>1.46<br>(1.28-1.64)	31<br>1.34<br>(1.08-1.7)	31<br>1.57<br>(1.36-1.75)	<0.001		0.013	
**Lipid-standardized[a] Vitamin E (VitEnorm, µmol/l)**	11<br>28.45<br>(25.31-32.7)	33<br>31.5<br>(27.7-33.6)	31<br>31.1<br>(28.1-33.8)	31<br>31.6<br>(28.4-33.5)				
**Homocysteine (Hcy, µmol/l)**	15<br>12.1<br>(10.1-16.1)	34<br>13.15<br>(10.1-15.3)	30<br>12.7<br>(10.9-15.3)	31<br>13<br>(9.9-18.2)				

	NW subjects	OB at T0	OB at T6	OB at T12	OB at T0 vs NW	OB<br>T0 vs T6	OB<br>T6 vs T12	OB<br>T0 vs T12
	n<br>*Median*<br>*(IQ range)*	*n*<br>*Median*<br>*(IQ range)*	*n*<br>*Median*<br>*(IQ range)*	*n*<br>*Median*<br>*(IQ range)*	*p-value**	*p-value***	*p-value***	*p-value***
**Cysteine**<br>**(Cys, µmol/l)**	15<br>270<br>(234-283)	34<br>315.5<br>(289-343)	30<br>315<br>(274-356)	31<br>330<br>(293-362)	<0.001			
**Cysteinylglycine**<br>**(CysGly, µmol/l)**	15<br>33<br>(31-40)	34<br>41.5<br>(35-47)	30<br>35.5<br>(30-40)	31<br>34<br>(32-40)	0.013	<0.001		0.002
**Glutathione**<br>**(Glu, µmol/l)**	13<br>7.1<br>(5.6-12.2)	32<br>6.85<br>(4.85-8.5)	26<br>7.35<br>(5.4-8.6)	27<br>7.8<br>(5.5-9.6)			0.033	
**^{1}H NMR serum metabolic markers**								
**Formic acid**	10<br>2.41<br>(1.77-2.67)	14<br>2.58<br>(2.45-2.76)	14<br>2.64<br>(2.49-2.87)	14<br>2.34<br>(1.87-2.79)				
**Glucose**	10<br>6.62<br>(5.82-7.93)	14<br>7.61<br>(6.85-8.23)	14<br>7.45<br>(6.55-7.8)	14<br>6.53<br>(5.24-7.02)	0.047			0.036
**Lactate**	10<br>25.75<br>(21.59-27.6)	14<br>34.91<br>(27.49-41.36)	14<br>33.89<br>(29.59-43.2)	14<br>34.2<br>(29.87-40.01)	0.010			
**Alanine**	10<br>48.7<br>(39.72-52.64)	14<br>46.74<br>(41.83-51.82)	14<br>45.22<br>(40.95-52.81)	14<br>35.16<br>(27.87-42.45)			0.006	0.001
**Choline containing metabolites (TMAO)**	10<br>52.55<br>(47.8-60.1)	14<br>49.56<br>(46.6-53.4)	14<br>50.23<br>(48.3-53.3)	14<br>48.82<br>(44.8-52.1)				
**L1**<br>**(Lipoprotein,**<br>**C*H3*-(CH2)n-)**	10<br>194.5<br>(189.8-210.2)	14<br>206.6<br>(200.2-210.6)	14<br>204.5<br>(201.4-218.1)	14<br>231.1<br>(205.6-236.7)			0.019	0.005
**L2**<br>**(Lipoprotein,**<br>**CH3−(C*H2*)n−)**	10<br>217.7<br>(183.4-225.1)	14<br>227.6<br>(219.2-241.8)	14<br>222.7<br>(218.5-239.5)	14<br>208.9<br>(191.3-230.3)	0.026		0.036	0.030
**L3**<br>**(fatty acids, FA**<br>**−C*H2*−CH2−C=O)**	10<br>131.6<br>(100.8-138.8)	14<br>151.5<br>(142.8-156.0)	14<br>155.7<br>(145.9-160.0)	14<br>161.6<br>(159.6-169.0)	<0.001			0.011
**L4**<br>**(monounsaturated FA;**<br>**MUFA, −C*H2*−CH=CH−)**	10<br>52.01<br>(44.3-161.5)	14<br>45.45<br>(43.9-47.3)	14<br>44.66<br>(42.5-49.5)	14<br>43.59<br>(41.6-44.6)	0.047			
**L5**<br>**(polyunsaturated FA;**<br>**PUFA, =CH−C*H2*−CH=)**	10<br>57.86<br>(47.5-66.6)	14<br>64.68<br>(61.9-66.6)	14<br>67.54<br>(62.4-71.2)	14<br>70.47<br>(66.2-77.7)				0.019
**L6**<br>**(unsaturated FA;**<br>**UFA, −C*H*=C*H*−)**	10<br>17.68<br>(14.19-20.21)	14<br>19.97<br>(18.54-23.29)	14<br>20.44<br>(18.1-23.39)	14<br>21.46<br>(18.55-25.44)				
**Total Cholesterol**	10<br>30.6<br>(24.75-36.92)	14<br>38.39<br>(34.33-41.47)	14<br>37.54<br>(34.66-42.11)	14<br>51.16<br>(44.26-58.78)	0.035		0.003	0.006
**Plasma peptide mediators**								
**CCL11^**<br>**(Eotaxin pg/ml)**	12<br>653.7<br>(319.8-892.9)	15<br>535.7<br>(374.4-632.6)	4<br>590.83<br>(412.9-635.4)	10<br>117.9<br>(102.4-627.2)				
**MCP-1/CCL2**<br>**(Monocyte Chemoattractant Protein-1; pg/ml)**	15<br>217.1<br>(168.5-276.4)	30<br>309.1<br>(261.5-388.5)	22<br>321.4<br>(229.4-375.8)	23<br>219.3<br>(173.6-310.5)	0.004		<0.001	<0.001
**MCP-2/CCL8**<br>**(Monocyte Chemoattractant Protein-2; pg/ml)**	14<br>48.65<br>(37.57-67.17)	30<br>54.31<br>47.43-67.11)	22<br>50.29<br>(36.53-62.13)	23<br>46.57<br>(33.14-68.21)				
**MCP-4/CCL13 (Monocyte Chemoattractant Protein-4; pg/ml)**	15<br>146.7<br>(117.9-194.8)	30<br>247.5<br>(153.2-360.2)	22<br>279.9<br>(195.4-424.3)	23<br>74.0<br>(42.2-326.2)	0.034			0.028
**IP-10/CXCL10**<br>**(Interferon γ-induced protein 10; pg/ml)**	15<br>34.15<br>(26.76-41.8)	30<br>52.9<br>(40.66-83.67)	22<br>57.22<br>(34.56-98.03)	23<br>36.09<br>(23.56-58.22)	0.002		0.035	0.022
**GROβ/CXCL2**<br>**(Growth-Regulated Protein Beta; pg/ml)**	15<br>282.1<br>(130.4-438.6)	30<br>231.2<br>(158.4-359.5)	19<br>244.9<br>(155.7-426.8)	21<br>196.8<br>(96.2-332.0)				

	NW subjects	OB at T0	OB at T6	OB at T12	OB at T0 vs NW	OB T0 vs T6	OB T6 vs T12	OB T0 vs T12
	n<br>*Median*<br>*(IQ range)*	*n*<br>*Median*<br>*(IQ range)*	*n*<br>*Median*<br>*(IQ range)*	*n*<br>*Median*<br>*(IQ range)*	*p-value**	*p-value***	*p-value***	*p-value***
**GM-CSF^ (Granulocyte-macrophage colony-stimulating factor; pg/ml)**	11<br>16.09<br>(10.32-20.61)	25<br>12.31<br>(3.29-25.02)	18<br>16.75<br>(4.29-41.15)	11<br>9.85<br>(6.3-26.05)				
**IL-1α (Interleukin-1α; pg/ml)**	14<br>35.63<br>(23.26-47.55)	30<br>44.50<br>(27.6-60.55)	22<br>30.86<br>(21.72-48.81	23<br>37.83<br>((27.13-60.55)				
**IL-6Lum (Interleukin-6; pg/ml)**	10<br>1.46<br>(1.2-2.19)	29<br>2.13<br>(1.39-2.86)	20<br>0.98<br>(0.49-1.5)	13<br>1.03<br>(0.53-1.6)		0.002		0.028
**TNF-αLum^ (Tumor necrosis factor- α; pg/ml)**	12<br>6.02<br>(5.35-7.33)	19<br>9.04<br>(7.17-11.42)	18<br>9.12<br>(7.49-10.1)	19<br>7.65<br>(5.52-8.74)	0.002		0.005	0.005
**IL-8/CXCL8^ (Interleukin-8; pg/ml)**	11<br>2.25<br>(1.36-6.67)	25<br>3.31<br>(1.81-5.38)	16<br>4.47<br>(2.86-7.27)	13<br>5.38<br>(1.73-6.33)				
**InsCPep (Insulin C Peptide; ng/ml)**	15<br>0.63<br>(0.49-0.81)	30<br>1.46<br>(1.11-2.0)	22<br>0.84<br>(0.57-1.15)	23<br>0.92<br>(0.82-1.22)	<0.001	<0.001		<0.001
**Leptin (ng/ml)**	15<br>10.7<br>(4.0-16.1)	30<br>87.0<br>(62.4-140.3)	22<br>26.6<br>(12.9-40.4)	23<br>17.0<br>(7.6-25.0)	<0.001	<0.001	0.015	<0.001
**Adiponectin (ng/ml)**	5<br>4.81<br>(3.09-7.11)	30<br>3.71<br>(2.59-5.48)	22<br>5.66<br>(3.94-8.06)	14<br>6.28<br>(4.44-8.52)		<0.001	0.016	0.002
**MMP-1 (Matrix Metalloproteinase-1; pg/ml)**	15<br>258.1<br>(180.9-378.7)	30<br>288.0<br>(235.3-427.4)	22<br>269.2<br>(220.2-357.3)	23<br>279.6<br>(169.6-687.9)				
**MMP-2 (Matrix Metalloproteinase-2; ng/ml)**	14<br>115.7<br>(94.1-12.4)	30<br>138.7<br>(124.6-165.8)	22<br>157.2<br>(144.5-164.9)	23<br>136.4<br>(117.4-153.8)	<0.001	0.042	0.040	
**MMP-3 (Matrix Metalloproteinase-3; ng/ml)**	14<br>8.51<br>(6.01-12.03)	30<br>10.03<br>(8.55-12.54)	22<br>9.05<br>(7.23-11.65)	23<br>9.47<br>(7.74-12.82)				0.036
**MMP-7 (Matrix Metalloproteinase-7; ng/ml)**	14<br>0.65<br>(0.54-0.87)	30<br>1.41<br>(1.02-2.15)	22<br>1.68<br>(1.39-2.33)	23<br>1.11<br>(0.68-2.02)	<0.001	0.039	0.001	
**MMP-9 (Matrix Metalloproteinase-9; ng/ml)**	14<br>42.1<br>(32.8-56.1)	30<br>83.1<br>(56.8-118.2)	22<br>48.3<br>(30.1-105.7)	23<br>36.1<br>(25.5-78.2)	<0.001			0.034
**MMP-10 (Matrix Metalloproteinase-10; pg/ml)**	15<br>516.8<br>(345.8-955.0)	30<br>553.6<br>(459.2-709.6)	22<br>514.5<br>(423.9-814.4)	23<br>653.5<br>(492.2-842.4)				
**EGF (Epidermal Growth Factor; pg/ml)**	8<br>6.53<br>(3.74-10.70)	30<br>6.23<br>(5.15-9.12)	22<br>7.58<br>(4.56-10.33)	19<br>7.64<br>(3.48-11.89)				
**PDGF-AA (Platelet-derived growth factor-AA; pg/ml)**	14<br>180.8<br>(47.6-318.5)	30<br>231.3<br>(143.6-344.3)	22<br>208.8<br>(113.6-271.6)	23<br>145.2<br>(81.3-276.6)				
**PDGF-BB (Platelet-derived growth factor-BB; pg/ml)**	14<br>505.9<br>(317.6-952.5)	30<br>519.8<br>(385.3-784.9)	22<br>580.6<br>(303.6-719.2)	23<br>460.3<br>(267.0-767.0)				
**VEGF-C (Vascular endothelial growth factor C; ng/ml)**	5<br>1.87<br>(1.60-2.08)	30<br>1.39<br>(0.94-1.84)	22<br>1.72<br>(1.13-1.94)	14<br>1.75<br>(0.80-2.14)				
**Red blood cell membrane fatty acids**								
**Myristic acid C14:0**	15<br>0.36<br>(0.21-0.48)	36<br>0.30<br>(0.25-0.42)	33<br>0.29<br>(0.21-0.39)	32<br>0.31<br>(0.23-0.38)				
**Palmitic acid C16:0**	15<br>21.85<br>(21.36-22.32)	36<br>22.89<br>(22.28-23.66)	33<br>22.88<br>(21.72-23.62)	32<br>22.26<br>(21.62-23.41)	0.001			0.009
**Sapienic acid C16:1;6c**	15<br>0.30<br>(0.19-0.42)	36<br>0.26<br>(0.17-0.44)	33<br>0.30<br>(0.20-0.63)	32<br>0.26<br>(0.18-0.46)				
**Palmitoleic acid C16:1;9c**	15<br>0.25<br>(0.19-0.42)	36<br>0.43<br>(0.29-0.59)	33<br>0.42<br>(0.30-0.54)	32<br>0.30<br>(0.24-0.41)	0.006			

	NW subjects	OB at T0	OB at T6	OB at T12	OB at T0 vs NW	OB<br>T0 vs T6	OB<br>T6 vs T12	OB<br>T0 vs T12
	n<br>*Median*<br>*(IQ range)*	*n*<br>*Median*<br>*(IQ range)*	*n*<br>*Median*<br>*(IQ range)*	*n*<br>*Median*<br>*(IQ range)*	*p-value**	*p-value***	*p-value***	*p-value***
**Heptadecanoic acid<br>C17:0**	15<br>0.35<br>(0.29-0.46)	36<br>0.30<br>(0.23-0.39)	33<br>0.33<br>(0.21-0.38)	32<br>0.33<br>(0.23-0.47)				
**Stearic acid<br>C18:0**	15<br>16.38<br>(15.87-16.86)	36<br>16.80<br>(16.16-17.14)	33<br>17.12<br>(15.66-17.72)	32<br>17.21<br>(16.21-18.08)				
**Trans Elaidate<br>C18:1; 9t**	15<br>0<br>(0-0.10)	36<br>0.58<br>(0-0.12)	33<br>0.58<br>(0.25-0.10)	32<br>0.51<br>(0.01-0.10)				
**Oleic acid<br>C18:1;9c**	15<br>17.36<br>(16.80-17.89)	36<br>17.40<br>(16.76-18.10)	33<br>17.97<br>(17.53-19.20)	32<br>18.15<br>(17.37-18.85)		<0.001		<0.001
**Vaccenic acid<br>C18:1;11c**	15<br>1.60<br>(1.40-1.86)	36<br>1.30<br>(1.15-1.54)	33<br>1.44<br>(1.21-1.72)	32<br>1.32<br>(1.06-1.57)	0.010			
**Linoleic acid<br>(LA)<br>C18:2; ω6**	15<br>13.71<br>(12.22-15.11)	36<br>12.08<br>(11.08-13.58)	33<br>11.08<br>(10.40-11.98)	32<br>11.90<br>(11.18-12.85)	0.006	<0.001	0.001	
**Dihomo-γ-linolenic acid<br>(DGLA)<br>C20:3; ω6**	15<br>1.69<br>(1.54-1.96)	36<br>2.39<br>(2.20-2.61)	33<br>1.80<br>(1.62-1.93	32<br>1.93<br>(1.74-2.18)	<0.001	<0.001		<0.001
**Arachidonic acid<br>(AA)<br>C20:4; ω6**	15<br>18.42<br>(16.60-19.70)	36<br>18.51<br>(17.49-19.29)	33<br>19.32<br>(18.70-20.60)	32<br>19.10<br>(17.54-20.02)		<0.001	0.030	0.032
**Mono-trans arachidonic acid (tAA)<br>C20:4; ω6**	15<br>0.00<br>(0.00-0.14)	36<br>0.04<br>(0-0.08)	33<br>0.06<br>(0.02-0.10)	32<br>0.03<br>(0.01-0.09)				
**Eicosapentaenoic acid<br>(EPA)<br>C20:5; ω3**	15<br>0.54<br>(0.40-0.77)	36<br>0.44<br>(0.30-0.61)	33<br>0.30<br>(0.25-0.51)	32<br>0.38<br>(0.26-0.61)		0.027		
**Docosapentaenoic acid<br>(DPA)<br>C22:5; ω3**	15<br>2.07<br>(1.71-2.18)	36<br>1.80<br>(1.59-1.98)	33<br>1.83<br>(1.68-2.09)	32<br>1.87<br>(1.60-2.10)				
**Docosahexaenoic acid<br>(DHA)<br>C22:6; ω3**	15<br>5.27<br>(3.42-5.86)	36<br>4.14<br>(3.37-4.72)	33<br>4.31<br>(3.57-4.91)	32<br>3.85<br>(3.30-4.73)				
**Saturated fatty acids<br>(SFA)**	15<br>38.91<br>(38.20-39.99)	36<br>40.48<br>(39.84-41.26)	33<br>40.32<br>(39.29-40.87)	32<br>40.27<br>(39.57-41.17)	<0.001			
**Monounsaturated fatty acids<br>(MUFA)**	15<br>19.39<br>(18.97-20.41)	36<br>19.73<br>(18.76-20.50)	33<br>20.29<br>(19.71-21.48)	32<br>20.13<br>(19.46-21.07)		<0.001	0.050	0.006
**ω6 polyunsaturated fatty acids<br>(ω6 PUFA)**	15<br>33.92<br>(32.84-35.30)	36<br>33.65<br>(31.91-34.36)	33<br>32.55<br>(31.53-33.47)	32<br>32.96<br>(31.77-33.99)				
**ω3 polyunsaturated fatty acids<br>(ω3 PUFA)**	15<br>7.50<br>(5.86-8.84)	36<br>6.39<br>(5.57-7.27)	33<br>6.83<br>(5.93-7.19)	32<br>5.99<br>(5.42-7.23)				
**Total Polyunsaturated fatty acids<br>(PUFA)**	15<br>41.24<br>(40.29-41.86)	36<br>39.64<br>(38.71-40.74)	33<br>39.41<br>(37.98-40.16)	32<br>39.15<br>(38.44-40.54)	0.002			
**SFA/MUFA Ratio**	15<br>2.02<br>(1.87-2.08)	36<br>2.06<br>(1.97-2.16)	33<br>2.03<br>(1.86-2.08)	32<br>1.99<br>(1.92-2.12)		0.004		0.021
**ω6/ω3 Ratio**	15<br>4.52<br>(3.93-6.03)	36<br>5.27<br>(4.34-6.19)	33<br>4.89<br>(4.35-5.48)	32<br>5.36<br>(4.34-6.37)			0.014	
**Total Trans fatty acids**	15<br>0.1<br>(0-0.22)	36<br>0.1<br>(0.05-0.18)	33<br>0.12<br>(0.08-0.17)	32<br>0.1<br>(0.05-0.17)				

* Mann-Whitney test. ** Wilcoxon signed-rank test. ^Variables not included in the Principal Component Analysis. [a]Vitamin A and E levels were standardized to 200 mg/dL (5.17 mmol/L) cholesterol and 110 mg/dL (1.24 mmol/L) triglycerides according to a multiple regression-based method.[8] Data of ^{1}H NMR serum metabolic markers are expressed as ‰ metabolite/all metabolites. Data of red blood cell membrane fatty acid are expressed as % fatty acid /all fatty acids.

Table S2. Principal component analysis: loading matrices of the main PCs extracted from each data set

Variable	PC1	PC2	PC3	PC4	PC5	PC6	PC7	PC8	PC9
**Clinical and clinical biochemistry markers (Clin)**									
**Weight**	**0,825**	-0,279	-0,235	0,036	-0,278	0,000	-0,107	0,119	-0,057
**Height**	0,147	-0,083	-0,024	**0,566**	**-0,374**	-0,219	-0,003	0,324	-0,302
**Body mass index (BMI)**	**0,822**	-0,260	**-0,275**	-0,269	-0,108	0,134	-0,102	-0,033	0,069
**Waist Circumference (WC)**	**0,844**	-0,146	-0,199	0,076	0,005	0,091	-0,227	0,161	-0,016
**Hip Circumference (HC)**	0,707	-0,255	**-0,377**	**-0,397**	-0,170	0,030	-0,015	0,011	0,047
**Waist-Hip ratio**	0,368	0,091	0,153	**0,546**	0,212	0,113	-0,316	0,232	-0,103
**Glycemia**	0,656	0,262	-0,176	0,179	**0,371**	-0,193	0,172	0,004	-0,113
**Insulin**	**0,841**	0,007	-0,140	-0,106	0,252	0,171	0,114	0,030	0,009
**HOMA-IR**	**0,839**	0,094	-0,162	-0,053	**0,334**	0,070	0,170	-0,007	-0,017
**HbA1c**	0,523	0,307	-0,257	0,215	**0,444**	-0,226	0,206	-0,044	-0,031
**Uric acid**	0,515	-0,223	0,258	0,279	-0,297	-0,003	-0,133	-0,186	0,251
**Creatinine**	0,335	-0,289	0,151	**0,399**	-0,169	0,164	**0,320**	-0,039	**0,371**
**Blood urea nitrogen**	0,419	0,242	0,295	**0,437**	-0,068	**0,436**	-0,075	-0,196	-0,169
**Red Blood Cells (RBC)**	0,337	0,228	0,011	0,307	-0,189	-0,139	**0,364**	**-0,491**	-0,144
**Platelets**	0,126	-0,260	0,373	**-0,546**	**0,383**	-0,254	-0,095	0,159	0,132
**Lymphocytes**	0,244	0,135	**0,656**	0,103	0,171	-0,324	-0,220	-0,276	-0,132
**Monocytes**	0,506	-0,215	**0,535**	0,086	0,292	-0,095	-0,287	0,079	0,034
**Eosinophils**	0,186	-0,386	0,322	0,269	0,263	0,310	0,096	0,204	0,106
**Basophils**	-0,010	-0,319	**0,514**	0,082	0,059	-0,238	0,275	-0,018	0,190
**Neutrophils**	0,421	-0,535	0,307	-0,076	0,133	-0,153	0,085	-0,090	0,020
**Total Cholesterol (TChol)**	-0,075	**0,842**	-0,107	0,143	0,182	0,001	-0,014	0,054	**0,324**
**HDL Cholesterol (HDL)**	-0,359	0,169	0,163	-0,003	0,309	**0,609**	**0,357**	0,147	0,052
**Triglycerides**	0,475	**0,594**	-0,168	-0,086	0,018	-0,045	**-0,326**	0,002	0,078
**LDL Cholesterol (LDL)**	-0,044	**0,762**	-0,173	0,210	0,094	-0,243	-0,058	0,003	**0,363**
**Aspartate Aminotransferase (AST)**	0,270	0,412	0,336	-0,254	**-0,431**	-0,124	0,260	0,079	-0,094
**Alanine transaminase (ALT)**	0,583	0,384	0,310	-0,272	-0,225	0,056	0,294	0,234	-0,210
**γ-Glutamyl Transferase (GGT)**	0,512	**0,582**	0,335	-0,143	-0,168	0,045	0,018	0,137	-0,177
**Alkaline Phosphatase (ALP)**	0,238	0,421	0,412	**-0,414**	-0,189	-0,015	0,090	0,226	0,190
**C-reactive protein, high sensitivity (hsCRP)**	**0,724**	-0,206	-0,164	-0,143	-0,041	-0,034	0,160	-0,274	0,193
**TNF-α**	0,072	-0,143	-0,118	**0,401**	**-0,326**	-0,258	0,102	**0,371**	**0,377**
**IL-6**	0,151	0,307	0,273	-0,131	-0,300	**0,344**	**-0,359**	-0,255	0,247
**Plasma oxidative stress markers (Ox)**									
**Advanced Oxidation Protein Products (AOPP)**	0,299	**-0,759**	**0,357**	0,092	0,090	-	-	-	-
**Total free thiols (SH)**	**0,784**	0,163	**-0,382**	0,390	0,081	-	-	-	-
**Total proteins**	**0,692**	**0,463**	0,065	0,132	-0,080	-	-	-	-
**SH normalized on total proteins (SHp)**	0,515	-0,174	**-0,602**	**0,430**	0,190	-	-	-	-
**Malondialdehyde (MDA)**	0,136	**-0,680**	**0,349**	0,093	0,087	-	-	-	-
**Vitamin A (VitA)**	**0,857**	-0,150	-0,111	-0,350	-0,078	-	-	-	-
**Vitamin E (VitE)**	**0,557**	0,008	**0,613**	0,431	-0,029	-	-	-	-
**Lipid-standardized Vitamin A (VitAnorm)**	**0,665**	-0,053	-0,352	**-0,558**	-0,032	-	-	-	-
**Lipid-standardized Vitamin E (VitEnorm)**	0,133	**0,715**	0,307	0,337	-0,039	-	-	-	-

Variable	PC1	PC2	PC3	PC4	PC5	PC6	PC7	PC8	PC9
**Homocysteine (Hcy)**	0,122	0,321	0,214	**-0,458**	**0,511**	-	-	-	-
**Cysteine (Cys)**	**0,564**	0,358	**0,480**	**-0,350**	-0,203	-	-	-	-
**Cysteinylglycine (CysGly)**	0,307	-0,305	0,175	-0,015	**0,577**	-	-	-	-
**Glutathione (Glu)**	-0,263	**0,463**	-0,024	0,054	**0,613**	-	-	-	-
**^{1}H NMR serum metabolic markers (Met)**									
**Formic acid**	-0,363	**0,745**	0,033	0,116	-	-	-	-	-
**Glucose**	-0,042	-0,165	0,284	**0,933**	-	-	-	-	-
**Lactate**	0,147	0,573	**-0,634**	**0,449**	-	-	-	-	-
**Alanine**	**-0,877**	0,331	0,262	0,080	-	-	-	-	-
**Choline containing metabolites (TMAO)**	-0,149	**0,743**	-0,086	0,166	-	-	-	-	-
**L1 (Lipoprotein, C*H3*-(CH2)n-)**	**0,882**	-0,125	0,032	-0,167	-	-	-	-	-
**L2 (Lipoprotein,CH3−(C*H2*)n−)**	-0,703	-0,489	-0,471	-0,106	-	-	-	-	-
**L3 (Fatty acids, −C*H2*−CH2−C=O)**	**0,858**	0,295	-0,273	0,001	-	-	-	-	-
**L4 (MUFA, −C*H2*−CH=CH−)**	-0,410	0,341	**0,775**	-0,244	-	-	-	-	-
**L5 (PUFA, =CH−C*H2*−CH=)**	0,701	0,460	**0,500**	-0,041	-	-	-	-	-
**L6 (UFA, −C*H*=C*H*−)**	0,310	**-0,713**	**0,417**	0,425	-	-	-	-	-
**Total cholesterol**	0,562	0,234	0,084	-0,036	-	-	-	-	-
**Plasma peptide mediators (Pep)**									
**CCL2/MCP1**	**-0,614**	0,343	**0,386**	-0,042	0,167	0,239	-	-	-
**CCL8/MCP2**	0,536	-0,321	**0,532**	0,064	0,121	0,069	-	-	-
**CCL13/MCP4**	-0,399	0,334	**0,620**	0,044	0,364	-0,048	-	-	-
**CXCL10/IP-10/CRG-2**	0,057	0,461	0,300	0,075	**0,513**	**-0,404**	-	-	-
**CXCL2/GROβ/MIP-2/CING-3**	-0,187	0,418	-0,170	**-0,345**	-0,370	0,345	-	-	-
**IL-1α**	**0,727**	-0,510	0,195	0,046	-0,098	0,195	-	-	-
**IL-6Lum**	0,294	-0,228	-0,130	-0,274	-0,060	**-0,342**	-	-	-
**Insulin C Peptide**	0,480	-0,224	0,146	**-0,460**	**0,406**	0,195	-	-	-
**Leptin**	0,155	-0,195	-0,244	0,056	0,309	0,094	-	-	-
**Adiponectin**	-0,006	-0,204	-0,144	**0,729**	-0,350	-0,216	-	-	-
**MMP-1**	0,364	0,497	-0,286	-0,060	-0,024	**0,491**	-	-	-
**MMP-2**	0,056	0,090	0,045	**0,816**	0,167	0,110	-	-	-
**MMP-3**	0,303	-0,209	**0,744**	0,110	-0,357	0,059	-	-	-
**MMP-7**	-0,365	0,340	**0,399**	0,035	-0,110	0,291	-	-	-
**MMP-9**	0,367	-0,136	**0,643**	-0,200	-0,330	-0,078	-	-	-
**MMP-10**	0,100	-0,153	0,013	**0,423**	0,173	**0,517**	-	-	-
**EGF**	**0,650**	**0,713**	-0,086	0,097	0,009	-0,073	-	-	-
**PDGF-AA**	**0,702**	**0,683**	-0,039	0,060	-0,039	-0,060	-	-	-
**PDGF-BB**	**0,671**	**0,714**	-0,062	0,053	0,008	-0,068	-	-	-
**VEGFC**	-0,312	**0,684**	0,246	0,006	**-0,365**	-0,135	-	-	-
**Red blood cell membrane fatty acids (MFA)**									
**Myristic acid C14:0**	-0,234	0,125	-0,016	0,409	0,036	-	-	-	-
**Palmitic acid C16:0**	-0,246	0,169	**0,634**	-0,139	**0,509**	-	-	-	-
**Sapienic acid C16:1;6c**	-0,052	-0,310	0,045	0,340	-0,157	-	-	-	-
**Palmitoleic acid C16:1;9c**	0,212	-0,314	0,121	0,361	-0,030	-	-	-	-
**Heptadecanoic acid C17:0**	-0,027	0,028	0,132	0,160	-0,069	-	-	-	-

Variable	PC1	PC2	PC3	PC4	PC5	PC6	PC7	PC8	PC9
**Stearic acid C18:0**	0,217	0,191	0,413	-0,164	**-0,624**	-	-	-	-
**Trans Elaidate C18:1; 9t**	-0,004	0,006	0,366	**0,598**	-0,080	-	-	-	-
**Oleic acid C18:1;9c**	-0,376	**-0,836**	-0,116	-0,081	0,009	-	-	-	-
**Vaccenic acid C18:1;11c**	-0,343	-0,261	-0,069	-0,241	0,306	-	-	-	-
**Linoleic acid (LA) C18:2; ω6**	**0,592**	0,354	-0,319	0,267	0,397	-	-	-	-
**Dihomo-γ-linolenic acid (DGLA) C20:3; ω6**	0,168	0,006	-0,077	-0,034	**0,638**	-	-	-	-
**Arachidonic acid (AA) C20:4; ω6**	0,389	-0,290	-0,182	-0,208	**-0,624**	-	-	-	-
**Mono-trans arachidonic acid C20:4; ω6**	0,072	-0,218	0,099	**0,518**	0,043	-	-	-	-
**Eicosapentaenoic acid (EPA) C20:5; ω3**	**-0,501**	0,310	0,077	0,305	-0,063	-	-	-	-
**Docosapentaenoic acid (DPA) C22:5; ω3**	-0,398	0,160	-0,426	0,384	-0,092	-	-	-	-
**Docosahexaenoic acid (DHA) C22:6; ω3**	**-0,649**	0,473	-0,363	-0,099	-0,077	-	-	-	-
**Saturated fatty acids (SFA)**	-0,098	0,328	**0,914**	-0,171	-0,023	-	-	-	-
**Monounsaturated fatty acids (MUFA)**	-0,400	**-0,901**	-0,096	-0,026	0,056	-	-	-	-
**ω6 polyunsaturated fatty acids (ω6 PUFA)**	**0,876**	0,095	-0,447	0,069	0,045	-	-	-	-
**ω3 polyunsaturated fatty acids (ω3 PUFA)**	**-0,741**	0,492	-0,402	0,089	-0,102	-	-	-	-
**Total Polyunsaturated fatty acids (PUFA)**	0,415	0,451	**-0,765**	0,137	-0,024	-	-	-	-
**SFA/MUFA Ratio**	0,244	**0,843**	0,456	-0,060	-0,046	-	-	-	-
**ω6/ω3 Ratio**	**0,853**	-0,367	0,250	-0,039	0,095	-	-	-	-
**Total Trans fatty acids**	0,042	-0,134	0,362	**0,819**	-0,038	-	-	-	-

Bold values represent the variables with the highest loadings and that have been considered for PC interpretation.

Table S3. Biological meaning of the main PCs extracted from each data set, as inferred by the maximally loaded variables. Variables are grouped by their loading sign, positive (+) or negative (-). Var = explained variance. The component loading matrix is presented in Table S2.

PC name	Var	Definition	Maximally loaded variables	Notes
Clinical and clinical biochemistry markers (Clin) – Cumulative Var = 77%				
**PC1-Clin**	25%	Adiposity, insulin resistance and systemic inflammation	Insulin, HOMA-IR, WC, BMI, weight and hsCRP (+)	
**PC2-Clin**	13%	Hepatic cholesterol metabolism	TChol, LDL, triglycerides, γGT (+)	Neutrophils, eosinophils and basophils also contribute to this component with negative loadings, suggesting an underlying, potential inverse association between cholesterol metabolism and myelopoiesis
**PC3-Clin**	9%	Adiposity-related immune system modulation	Lymphocytes, monocytes and basophils (+)<br>HC and BMI (-)	
**PC4-Clin**	8%	Sex- and age-related body composition	Height, waist-to-hip ratio, blood urea nitrogen, TNF-α and creatinine (+)<br>Platelets, ALP and HC (-)	All of these variables are related to anthropometric and physiological features typical of male and/or older people (greater height, central fat (re)distribution, higher local inflammation indexes, higher muscle mass size and/or catabolism, low platelet count and decrease in bone mass turnover)
**PC5-Clin**	6%	Glucose metabolism-related thrombotic risk	HbA1c, platelets, glucose and HOMA-IR (+)<br>AST, height and TNF-α (-)	The variables with negative loadings, all characterized by higher values in men than in women, suggest that this component especially represents the thrombotic risk of female patients. This interpretation is supported by the evidence that in female but not in male patients, obesity is significantly associated with an increase in platelet number.[9]
**PC6-Clin**	5%	Muscle catabolism	HDL, blood urea nitrogen and IL-6 (+)	HDL levels are inversely correlated to non-adipose body mass,[10] blood urea nitrogen is a marker of protein catabolism, and IL-6 reflects the contribution of inflammation to muscle loss.
**PC7-Clin**	4%	Protein intake-related multisystem metabolism	RBC, HDL and creatinine (+)<br>IL-6 and triglycerides (-)	Such component represents the effects of protein intake on bone marrow production of red cells, on inflammation,[11] on hepatic lipid metabolism and serum creatinine balance.
**PC8-Clin**	4%	Local inflammation-related impaired erythropoiesis.	TNF-α (+)<br>RBC (-)	Such component pictures the effects of local inflammation on bone marrow production of red cells
**PC9-Clin**	3%	Local inflammation and dyslipidemia-related renal dysfunction	TNF-α, creatinine, LDL and TChol (+)	Such component pictures the effects of local inflammation and lipid abnormalities on renal function
Plasma oxidative stress markers (Ox) – Cumulative Var 78%				
**PC1-Ox**	27%	Nutritional status	VitA, total thiols, VitAnorm, total proteins, VitE and Cys (+)	Such component includes all the not normalized variables and it simply mirrors the nutritional intake
**PC2-Ox**	18%	Global oxidative balance	VitEnorm, Glu and total proteins (+)<br>AOPP and MDA (-)	Such component pictures the balance between antioxidant capacity and oxidative damage.
**PC3-Ox**	13%	Lipid-associated oxidative stress	VitE, Cys, AOPP and MDA (+)<br>SHp and SH (-)	Not normalized VitE levels are strongly correlated to plasma lipids (r(s) = 0.77 and 0.68, VitE vs total cholesterol and tryglicerides, respectively; n = 95, $p<0.001$). Hence, in this component we assume VitE as a lipid proxy. This component mirrors the positive association between lipid levels and oxidative stress, as represented by the specular contributions of oxidative damage markers and thiol redox status.
**PC4-Ox**	11%	Liver-related plasma redox status	SHp (+)<br>VitAnorm, Hcy and Cys (-)	This component mirrors a relationship between plasma thiol redox status and the liver function, the latter being represented by variables which plasma levels are all linked to liver metabolism.
**PC5-Ox**	8%	Glutathione metabolism	Glu, Hcy and CysGly (+)	These variables represent low-MW thiols involved in glutathione metabolism.

PC name	Var	Definition	Maximally loaded variables	Notes
		^{1}H NMR serum metabolic markers (Met) – Cumulative Var 84%		
**PC1-Met**	34%	Energy substrate balance	L1 (lipoproteins) and L3 (fatty acids) (+)<br>Alanine (-)	This component reflects an inverse relationship between two main energetic substrates, i.e., lipids on one hand, and the principal gluconeogenic aminoacid, alanine, on the other hand.
**PC2-Met**	23%	One-carbon metabolism	Formic acid, choline containing metabolites (TMAO) (+)<br>L6 (unsaturated fatty acids) (-)	The variables with positive loadings represent metabolites of one-carbon metabolism while the variables with a negative loading reflects the contribution of the breakdown of phosphatidylcholine (usually bearing unsaturated fatty acids) to choline availability.
**PC3-Met**	16%	Liver fatty acid desaturase activity	L4 (MUFA), L5 (PUFA) and L6 (UFA) (+)<br>Lactate (-)	This component mirrors the contribution of lactic acid consumption, via lactic dehydrogenase activity and NADH regeneration, to plasma fatty acid desaturation in liver. [12]
**PC4-Met**	12%	Glucose metabolism	Glucose and lactate (+)	Lactate is a main glucose metabolite. This component reflects glucose handling by peripheral tissues.[13]
		Plasma peptide mediators (Pep) – Cumulative Var 71%		
**PC1-Pep**	19%	Tissue reparative processes vs inflammatory macrophage infiltration	IL-1α, PDGF-AA, PDGF-BB and EGF (+)<br>MCP-1 (-).	This component describes the inverse relationship between reparative pro-fibrotic stimuli and active inflammation driven by monocyte/macrophage chemotaxis.[14,15]
**PC2-Pep**	18%	Angiogenic processes	VEGF-C, EGF, PDGF-AA and PDGF-BB (+)	
**PC3-Pep**	12%	Macrophage infiltration-driven extracellular matrix remodeling	MMP-3, MMP-9, MCP-4, MCP-2, MMP-7 and MCP-1 (+)	This component emphasizes the relationship between monocyte/macrophage recruitment, fat infiltration and the remodeling of extracellular matrix.[16]
**PC4-Pep**	9%	Adipose tissue function and insulin sensitivity	MMP-2, MMP-10 and adiponectin (+)<br>InsCPep and GROβ / CXCL2 (-)	This component reflects the connection between ECM remodeling and inflammation control mediated by MMPS and the metabolic functions of adipose tissue promoting insulin sensitivity.[17,18]
**PC5-Pep**	7%	Liver injury and insulin resistance	IP-10 and InsCPep (+)	IP-10 is a pro-inflammatory cytokine whose levels are increased in type 2 diabetic patients [19] and which is associated with progressive liver injury, insulin resistance and incident diabetes in mostly obese NAFLD patients.[20]
**PC6-Pep**	6%	Control of leukocytes activation	MMP-10 and MMP-1 (+)<br>IP-10 and IL-6 (-)	This component reflects the balance between the modulating effect of MMP-10[18,21] and MMP-1[22] and the stimulating effect of IP-10 and IL-6Lum on leukocyte activation
		RBC membrane fatty acids (MFA) – Cumulative Var 65%		
**PC1-MFA**	18%	Omega-6 / omega-3 fatty acids competition	Omega-6/omega-3 ratio, omega-6 PUFA and omega-6 linoleic acid (+)<br>Omega-3 PUFA, omega-3 EPA and omega-3 DHA (-)	This component represents the competition between omega-6 and omega-3 fatty acids, an index mirroring the nutritional status and inked with the pro- and anti-inflammatory potential of the cell membrane.
**PC2-MFA**	16%	Saturated to monounsaturated fatty acid ratio	SFA/MUFA (+)<br>MUFA and oleic acid (-)	This component represents the SFA/MUFA ratio and mirrors the opposite of MUFA biosynthesis.
**PC3-MFA**	14%	De novo lipogenesis - saturated to polyunsaturated fatty acids ratio	SFA and palmitic acid (+)<br>PUFA (+)	This component represents the SFA/PUFA ratio and mirrors the inverse relationship between the de novo lipogenesis (a metabolic index) and the diet-dependent fraction of polyunsaturated fatty acids in the membrane (a nutritional index).
**PC4-MFA**	10%	Free radical stress	Total trans fatty acids, trans elaidate and mono-trans arachidonic acid (+)	Trans fatty acids can be endogenously formed by free radical stress, which in turn is related to oxidative stress and inflammation in subjects affected by obesity.[23]
**PC5–MFA**	7%	Saturated and omega-6 fatty acids metabolism	DGLA and palmitic acid (+)<br>AA and stearic acid (-)	This component represents a cross-road of two fatty acid transformation routes, the exogenous omega-6 DGLA desaturation (delta5-desaturase) and the endogenous SFA palmitic acid elongation (elongase 6). The two substrates (DGLA and palmitic acid) enter in the component with a positive sign while the two metabolites (AA and stearic acid) with a negative one, suggesting that this component represents both enzymes' activities with a negative sign.

Figure S2. Temporal trends of PCs that are not significantly affected by bariatric surgery. PC values at baseline (T0), six (T6) and twelve (T12) months from BS are reported. Values are means with 95% CI. SFA = saturated fatty acids; PUFA = polyunsaturated fatty acids.

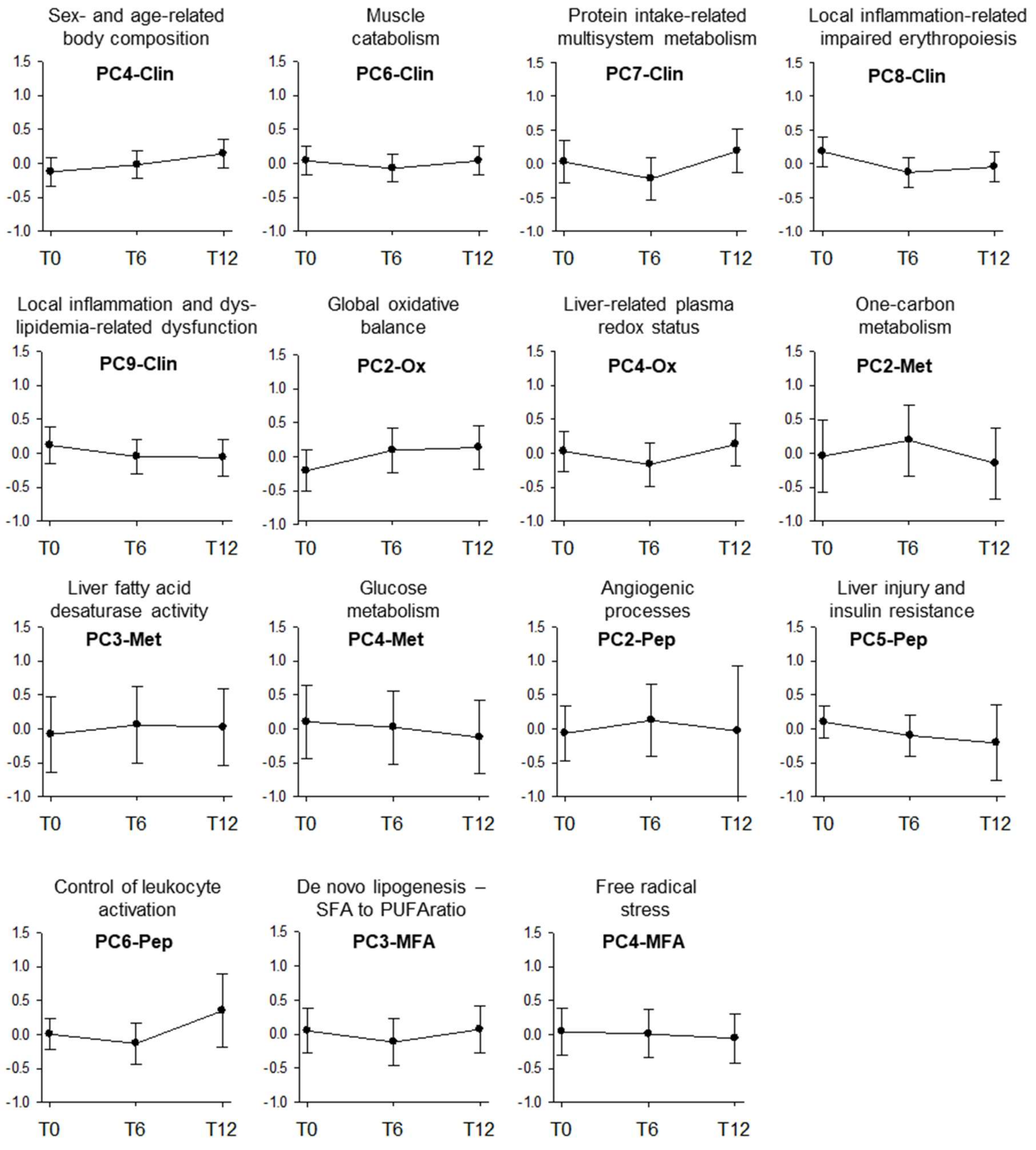

References

1. Giuliani, A. The application of principal component analysis to drug discovery and biomedical data. *Drug Discov. Today* **22**, 1069–1076 (2017).
2. Cattell, R. B. The Scree Test For The Number Of Factors. *Multivariate Behav. Res.* **1**, 245–276 (1966).
3. Härdle, W. K. & Simar, L. Canonical Correlation Analysis. in *Applied Multivariate Statistical Analysis* 443–454 (Springer Berlin Heidelberg, 2015). doi:10.1007/978-3-662-45171-7_16.
4. Sirabella, P., Giuliani, A., Colosimo, A. & Dippner, J. Breaking down the climate effects on cod recruitment by principal component analysis and canonical correlation. *Mar. Ecol. Prog. Ser.* **216**, 213–222 (2001).
5. Heffron, S. P. *et al.* Changes in Lipid Profile of Obese Patients Following Contemporary Bariatric Surgery: A Meta-Analysis. *Am. J. Med.* **129**, 952–959 (2016).
6. Strain, G. W. *et al.* Changes in lipid profiles in morbidly obese patients after laparoscopic sleeve gastrectomy (LSG). *Obes. Surg.* **21**, 305–309 (2011).
7. Panee, J. Monocyte Chemoattractant Protein 1 (MCP-1) in obesity and diabetes. *Cytokine* **60**, 1–12 (2012).
8. Jordan, P., Brubacher, D., Moser, U., Stahelin, H. B. & Gey, K. F. Vitamin E and vitamin A concentrations in plasma adjusted for cholesterol and triglycerides by multiple regression. *Clin. Chem.* **41**, 924–927 (1995).
9. Samocha-Bonet, D. *et al.* Platelet Counts and Platelet Activation Markers in Obese Subjects. *Mediators Inflamm.* **2008**, 1–6 (2008).
10. Pietrobelli, A., Lee, R. C., Capristo, E., Deckelbaum, R. J. & Heymsfield, S. B. An independent, inverse association of high-density-lipoprotein-cholesterol concentration with nonadipose body mass. *Am. J. Clin. Nutr.* **69**, 614–620 (1999).
11. Porter Starr, K. N. *et al.* Influence of Weight Reduction and Enhanced Protein Intake on Biomarkers of Inflammation in Older Adults with Obesity. *J. Nutr. Gerontol. Geriatr.* **38**, 33–49 (2019).
12. Kim, W. *et al.* Polyunsaturated Fatty Acid Desaturation Is a Mechanism for Glycolytic NAD+ Recycling. *Cell Metab.* **29**, 856-870.e7 (2019).
13. Lagarde, D. *et al.* Lactate Fluxes and Plasticity of Adipose Tissues: A Redox Perspective. *Front. Physiol.* **12**, (2021).
14. Marcelin, G., Gautier, E. L. & Clément, K. Adipose Tissue Fibrosis in Obesity: Etiology and Challenges. *Annu. Rev. Physiol.* **84**, (2022).
15. Zamarron, B. F. *et al.* Macrophage Proliferation Sustains Adipose Tissue Inflammation in Formerly Obese Mice. *Diabetes* **66**, 392–406 (2017).
16. Berg, G., Barchuk, M. & Miksztowicz, V. Behavior of Metalloproteinases in Adipose Tissue, Liver and Arterial Wall: An Update of Extracellular Matrix Remodeling. *Cells* **8**, 158 (2019).
17. Fingleton, B. Matrix metalloproteinases as regulators of inflammatory processes. *Biochim. Biophys. Acta - Mol. Cell Res.* **1864**, 2036–2042 (2017).
18. McMahan, R. S. *et al.* Stromelysin-2 (MMP10) Moderates Inflammation by Controlling Macrophage Activation. *J. Immunol.* **197**, 899–909 (2016).
19. Pan, X., Kaminga, A. C., Wen, S. W. & Liu, A. Chemokines in Prediabetes and Type 2 Diabetes: A Meta-Analysis. *Front. Immunol.* **12**, 1–17 (2021).
20. Chang, C. C. *et al.* Interferon gamma-induced protein 10 is associated with insulin resistance and incident diabetes in patients with nonalcoholic fatty liver disease. *Sci. Rep.* **5**, 1–11 (2015).
21. Rohani, M. G. *et al.* Macrophage MMP10 Regulates TLR7-Mediated Tolerance. *Front. Immunol.* **9**, 1–8 (2018).
22. Van Lint, P. & Libert, C. Chemokine and cytokine processing by matrix metalloproteinases and its effect on leukocyte migration and inflammation. *J. Leukoc. Biol.* **82**, 1375–1381 (2007).
23. Sinclair, H. Trans fatty acids and weight gain AK Thompson, A-M Minihane and CM Williams. *Int. J. Obes.* **35**, 315–324 (2011).
